# Acceptance of AI-assisted English language learning tools in higher education: psychological correlates across disciplinary and proficiency groups

**DOI:** 10.3389/fpsyg.2026.1806457

**Published:** 2026-07-01

**Authors:** Jin Wu, Yiyun Wang, Yun He, Xueqi Yin, Fang Chen, Bingrong Wan

**Affiliations:** 1 School of Foreign Languages, Tianjin University of Commerce, Tianjin, China; 2 School of Economics, Tianjin University of Commerce, Tianjin, China

**Keywords:** AI-assisted language learning, higher education context, learner psychology, learning motivation, self-efficacy, technology acceptance

## Abstract

Artificial intelligence (AI)-assisted language learning tools are increasingly used in higher education, yet student acceptance of these tools may vary across academic disciplines and English proficiency levels. Building on the Technology Acceptance Model (TAM) as a baseline framework, this study examined how learning motivation, self-efficacy, anxiety, and risk perception were associated with acceptance outcomes for AI-assisted English learning in a Chinese higher education context. The study focused on perceived usefulness, perceived ease of use, behavioral intention, and satisfaction, and further examined mean-level group differences and descriptive subgroup-specific association patterns by academic discipline and English proficiency. Survey data were collected from 210 undergraduates (STEM = 91, Humanities = 119; English proficiency: Low = 77, Intermediate = 103, High = 30). Data analyses were conducted using IBM SPSS Statistics 27.0 and Stata 18.0, including descriptive statistics, reliability and convergent validity analyses, full-sample adjusted linear regression models using robust standard errors, mean-level group comparisons, *post hoc* comparisons, and exploratory subgroup-specific regression analyses. Learning motivation and self-efficacy were consistently and positively associated with acceptance-related indicators. Anxiety did not show a uniformly negative pattern; its positive associations with acceptance outcomes were exploratory concurrent patterns and should not be interpreted as evidence that anxiety is beneficial. Risk perception was also positively but generally more weakly associated with acceptance outcomes and should be interpreted cautiously. Mean-level comparisons indicated descriptive differences in several acceptance outcomes across academic discipline and English proficiency groups. Exploratory subgroup-specific regressions further described within-group association patterns; these analyses were not formal tests of between-group differences in regression coefficients. Given the cross-sectional design, all findings should be interpreted as associative rather than causal. Overall, the study provides a learner-centered account of AI-assisted English learning acceptance by highlighting psychological correlates and group-related descriptive patterns in a Chinese higher education context.

## 1 Introduction

Artificial intelligence (AI) has become increasingly embedded in higher education, reshaping how students access learning resources, interact with digital systems, and receive instructional support. In policy and sector-level discussions, AI is often linked to broader agendas concerning personalization, learner autonomy, lifelong learning, and Education 4.0, as reflected in development-oriented frameworks promoted by international organizations such as UNESCO and the World Economic Forum (Shaheed, 2024; UNESCO, n.d.; World Economic Forum, 2020). AI in education has also been discussed in relation to wider social and educational objectives, including the Sustainable Development Goals articulated by the United Nations (United Nations General Assembly, 2015). At the technical and pedagogical levels, advances in machine learning, natural language processing, and large-scale language models have supported the expansion of AI-powered educational systems, including adaptive tutoring, automated feedback, personalized learning pathways, and learning analytics (Graesser et al., 2012; Zawacki-Richter et al., 2019).

Language education has been a prominent site for these developments. In computer-assisted language learning and English as a foreign language instruction, AI-assisted tools such as intelligent tutoring systems, chatbots, simulations, automated writing support, and adaptive platforms have been used to provide individualized practice, interactive feedback, and flexible learning support (Bayly-Castaneda et al., 2024; Chen, 2024; Létourneau et al., 2025; Shute and Zapata-Rivera, 2012; Tan et al., 2025; Zawacki-Richter et al., 2019). Recent studies have further extended this line of inquiry to AI-mediated informal digital learning of English (AI-IDLE). For example, Wang et al. (2025) used Q methodology to examine Chinese EFL learners’ beliefs about AI-mediated IDLE, highlighting the diversity of learner perspectives in AI-supported informal English learning contexts. Wang et al. (2026) further examined L2 self-guides, achievement emotions, and engagement in AI-mediated IDLE through structural equation modeling and psychological network analysis, indicating the growing attention to motivational and affective processes in AI-supported English learning. The rapid diffusion of large language model-based applications has further accelerated interest in AI-assisted English learning by enabling more flexible dialogue, explanation, revision, and personalized guidance at scale (Wei, 2023). Initiatives such as the “AI for Language Education” project from the European Centre for Modern Languages also reflect the growing institutional attention given to pedagogically grounded and ethically attentive AI implementation in language education (European Centre for Modern Languages [ECML], 2024).

Despite these developments, the availability of AI-assisted tools does not automatically lead to acceptance or sustained use. Students may perceive AI-supported learning as useful, convenient, and motivating, but they may also experience uncertainty, anxiety, skepticism, or concerns about privacy, authenticity, and academic integrity (Bewersdorff et al., 2025; Capiola et al., 2023; Chan, 2023; Favero et al., 2025; Huang, 2023; Kasneci et al., 2023; Khan et al., 2025; Selwyn, 2019a,b; Trust et al., 2023). In language learning contexts, these responses may be especially salient because learners are required not only to operate a technological system, but also to engage with feedback, evaluate linguistic suggestions, revise output, and manage uncertainty during communication-oriented tasks. Therefore, examining students’ acceptance of AI-assisted English learning tools in association with learner perceptions, psychological resources, affective and evaluative correlates, and learning contexts may complement system-focused accounts.

The Technology Acceptance Model (TAM) provides a well-established baseline for examining technology use through perceived usefulness (PU) and perceived ease of use (PEOU), which are commonly linked to acceptance-related outcomes such as behavioral intention (BI) (Davis, 1989; Fishbein and Ajzen, 1975). In educational technology research, TAM has been widely used to organize learners’ evaluations of whether a system is helpful, manageable, and likely to be used in the future (Granić and Marangunić, 2019; Scherer et al., 2019). In addition, post-use evaluative outcomes such as satisfaction have been used to capture learners’ overall appraisal of technology-supported learning experiences (Bhattacherjee, 2001). However, when applied to AI-assisted English learning, a purely system-centered or usability-centered account may be insufficient. Learners’ judgments of usefulness, ease of use, satisfaction, and behavioral intention may be associated with learner psychological resources and affective or evaluative correlates related to how AI-supported learning activities are experienced. Prior extensions of TAM have therefore incorporated constructs such as self-efficacy, motivation, anxiety, and perceived risk to better account for learner-centered correlates and context-sensitive constraints (Dishaw and Strong, 1999; Featherman and Pavlou, 2003; Šumak et al., 2011; Venkatesh, 2000; Venkatesh and Davis, 2000).

Several learner-centered variables are particularly relevant to AI-assisted English learning. Learning motivation may be associated with whether students perceive AI-supported practice, feedback, and revision as valuable for language development. Self-efficacy may be associated with whether students feel capable of managing AI-supported tasks, interpreting feedback, and using the tool to support learning goals (Bandura, 1986; Compeau and Higgins, 1995; Ryan and Deci, 2017). Anxiety is also relevant because AI-supported language learning may involve uncertainty, performance pressure, or concern about the quality of one’s output. However, anxiety should not be assumed to operate as a uniformly negative factor in all learning settings. From a control-value perspective, anxiety may vary in its associations with learning-related evaluations depending on whether tasks are perceived as meaningful and manageable (Pekrun, 2006; Scovel, 1978). Risk perception is another relevant factor, particularly in relation to privacy, data use, and the perceived consequences of relying on AI-mediated learning support (Dinev and Hart, 2006; Featherman and Pavlou, 2003; Ifenthaler and Schumacher, 2016; OECD, 2021). These variables provide a learner-centered basis for examining AI-assisted English learning acceptance without assuming that all psychological factors operate in the same direction or with the same strength.

Acceptance may also vary across learner groups in descriptive terms. Disciplinary background may be associated with differences in learning norms, epistemological orientations, interaction preferences, and expectations regarding technology-supported learning (Becher and Trowler, 2001; Fry and Talja, 2007). Similarly, English proficiency may be associated with how learners evaluate the usefulness, manageability, and affective demands of AI-assisted English learning tools. Lower-proficiency learners may require more scaffolding and may evaluate AI support differently from higher-proficiency learners, who may use AI tools for refinement, verification, or more advanced language production. Such group-related variation does not imply fixed causal effects of discipline or proficiency, but it provides a useful contextual basis for examining whether acceptance-related patterns appear uniform across learners.

Despite the growing literature on AI-assisted learning acceptance, three gaps remain particularly relevant to AI-assisted English learning in higher education. First, previous acceptance studies have often examined motivational, efficacy-related, affective, or risk-related variables separately, leaving limited evidence on how these psychological correlates, when considered together, relate to multiple acceptance outcomes within a single regression-based empirical framework. Second, compared with perceived usefulness and ease of use, anxiety and risk perception have received less sustained attention in AI-assisted English learning, especially as context-sensitive correlates rather than uniformly negative barriers. Third, evidence remains limited on whether acceptance-related association patterns show descriptive variation when examined within disciplinary backgrounds and English proficiency levels, as much existing acceptance research has tended to model learner populations as relatively homogeneous. Addressing these gaps may provide a more differentiated account of students’ acceptance of AI-assisted English learning tools without assuming that the same psychological factors operate uniformly across learner groups.

Against this background, the present study examines university students’ acceptance of AI-assisted English language learning tools in the Chinese higher education context using a cross-sectional survey design. Building on TAM as a baseline framework, the study investigates how learning motivation, self-efficacy, anxiety, and risk perception are associated with four acceptance outcomes: PU, PEOU, satisfaction, and BI. It also examines mean-level variation in acceptance outcomes across academic discipline and English proficiency groups and reports exploratory subgroup-specific association patterns estimated separately within these groups, without formal comparisons of regression coefficients. Given the cross-sectional design, the study is framed to identify patterns of association rather than to establish causal relationships or temporal processes.

### 1.1 Research questions and theoretically informed expectations

Based on the preceding review, this study addressed the following research questions, which were examined within an associational and exploratory framework without claims of causal inference:

RQ1. In the overall analytical sample (*N* = 210), to what extent and in what directions are self-efficacy, learning motivation, anxiety, and risk perception associated with perceived usefulness, perceived ease of use, satisfaction, and behavioral intention in AI-assisted English learning?

RQ2. Do the mean levels of perceived usefulness (PU), perceived ease of use (PEOU), satisfaction, and behavioral intention (BI) differ across academic discipline groups (Humanities and STEM) and English proficiency groups (Low, Intermediate, and High based on CET-4 scores)?

RQ3. What descriptive patterns of association between psychological correlates and acceptance outcomes are observed when exploratory adjusted regression analyses are estimated separately within academic discipline and English proficiency subgroups?

In addition to these research questions, two theoretically informed expectations were formulated for the overall sample, specifically concerning learning motivation and self-efficacy. First, informed by self-determination theory (Ryan and Deci, 2017), learning motivation was considered theoretically likely to show positive associations with the four acceptance outcomes, particularly perceived usefulness, satisfaction, and behavioral intention. This expectation was based on the view that learners who report higher motivation for AI-assisted English learning may be more likely to perceive such tools as valuable for language development. Second, drawing on social cognitive theory (Bandura, 1986), self-efficacy was considered theoretically likely to show positive associations with the four acceptance outcomes, particularly perceived ease of use and satisfaction. This expectation was aligned with theoretical reasoning suggesting that learners who feel more capable of managing AI-supported learning tasks may evaluate such interactions as more manageable and beneficial.

In contrast, associations involving anxiety and risk perception were treated as exploratory, given the limited and context-dependent evidence in AI-assisted English learning settings. The same exploratory orientation applied to the mean-level group comparisons and to the subgroup-specific association patterns related to academic discipline and English proficiency. No formal tests of between-subgroup differences in regression coefficients were conducted, and no directional expectations were formulated for these exploratory analyses.

The remainder of this article is organized as follows. The next section presents the theoretical background, including TAM-based acceptance constructs, learner psychological resources, affective and evaluative correlates, and disciplinary and proficiency-based variation in acceptance. The following sections describe the research design, measures, analytical procedures, and empirical findings. The final sections discuss the results, limitations, and implications for AI-assisted English learning in higher education.

## 2 Theoretical background and literature review

### 2.1 Technology acceptance in AI-assisted English learning

Technology acceptance research has frequently used the Technology Acceptance Model (TAM) as a parsimonious baseline framework for organizing key acceptance beliefs and acceptance-related indicators (Davis, 1989; Fishbein and Ajzen, 1975). In AI-assisted language learning, TAM offers a common set of terms for describing how learners judge instructional support and interaction feasibility and how these judgments may relate to intention and post-use evaluation (Granić and Marangunić, 2019; Scherer et al., 2019).

Within this baseline framing, perceived usefulness (PU) captures learners’ belief that AI-supported interaction contributes to learning progress and task accomplishment. In English learning contexts, PU can be understood as learning-relevant support, including perceived support for comprehension, accuracy, fluency, expression quality, and task completion, rather than as generic productivity. Perceived ease of use (PEOU) indexes the degree to which interaction with the tool is experienced as manageable, understandable, and not overly cognitively demanding during routine use. In AI-assisted English learning, PEOU includes learners’ perceptions of whether AI-supported interaction steps, feedback interpretation, and iterative revision processes are understandable and navigable.

Behavioral intention (BI) reflects learners’ intention to continue using AI-assisted tools in future learning activities. Satisfaction is treated as a post-use evaluative outcome reflecting learners’ overall judgment of the AI-supported learning experience after engagement (Bhattacherjee, 2001). Although satisfaction is not part of Davis’s original TAM, it has been widely adopted as an evaluative outcome in technology-mediated learning research. In AI-assisted learning, satisfaction captures learners’ retrospective evaluation of the technology, including how they experienced feedback quality, interaction flow, and instructional value.

Rather than offering a critique of or replacement for the baseline TAM framework, learner-centered psychological variables are introduced as complementary factors that may relate to acceptance outcomes. Accordingly, the present study retains PU, PEOU, BI, and satisfaction as baseline acceptance-related constructs, while examining learning motivation, self-efficacy, anxiety, and risk perception as theoretically informed or exploratory correlates.

### 2.2 Learner psychological resources: motivation and self-efficacy

Acceptance-related beliefs in AI-assisted English learning may also relate to the value and capability resources that learners bring to technology-supported learning activities. This section focuses on learning motivation and self-efficacy as two distinct learner psychological resources, rather than as indicators of an additional latent construct.

Learning motivation represents learners’ willingness to invest effort in language learning and the value-oriented reasons underlying that effort, including both intrinsic interest and extrinsic goal pursuit (Ryan and Deci, 2017). From the perspective of Self-Determination Theory, motivation addresses the value dimension of AI-assisted English learning. Learners who view English learning as meaningful or goal-relevant may be more likely to perceive AI feedback, guided practice, and adaptive learning support as useful for language development. Motivation may therefore be associated with PU, BI, and satisfaction.

Self-efficacy captures learners’ perceived capability to manage AI-supported learning tasks (Bandura, 1986, 1997; Compeau and Higgins, 1995). Grounded in Social Cognitive Theory, it addresses the capability and control dimension of technology-supported learning. Self-efficacy may be associated with PEOU, as learners who feel more capable of managing AI-supported tasks may experience the interaction as less demanding. It may also relate to satisfaction, particularly when learners feel able to interpret automated feedback, revise language output, and regulate the pace of AI-supported practice.

Learning motivation and self-efficacy therefore provide two complementary perspectives on learner psychological resources. Motivation addresses why effort may be worth investing, whereas self-efficacy addresses whether learners feel capable of sustaining that effort under task demands. In the present study, these constructs are examined as theoretically informed correlates in regression-based analyses, without being treated as indicators of a separate higher-order construct.

### 2.3 Affective and evaluative correlates: anxiety and risk perception

AI-assisted English learning may also involve affective and evaluative responses, specifically anxiety and risk perception, that relate to acceptance outcomes in context-sensitive ways.

Anxiety can be understood as a task-related affective state that may arise during AI-assisted English learning activities. From a Control-Value Theory perspective, achievement-related emotions are linked to learners’ appraisals of control over learning processes and the perceived value of the activity (Pekrun, 2006). In AI-assisted English learning, anxiety may arise when learners receive immediate automated feedback, compare their own output with AI-generated suggestions, or become aware of gaps in accuracy, fluency, or expression quality.

The present study does not assume that anxiety is uniformly negative or uniformly detrimental; rather, it is examined as a concurrent affective correlate. In some instructional contexts, anxiety may coexist with favorable acceptance indicators, particularly when learners also report stronger value-related and capability-related appraisals (Brown et al., 2023).

Risk perception captures learners’ subjective appraisal of potential costs associated with AI-mediated learning tools, especially concerns about data handling, privacy exposure, and uncertainty during routine learning use (Featherman and Pavlou, 2003; Ifenthaler and Schumacher, 2016). In educational settings, risk perception may be associated with acceptance-related outcomes as learners consider not only usefulness and ease of use but also potential costs or concerns.

The privacy calculus perspective provides a contextual lens for interpreting how perceived benefits and perceived costs may be considered in technology use (Dinev and Hart, 2006). In this study, privacy calculus is used as a descriptive framing rather than as a tested structural mechanism. Because institutional trust was not directly measured, it is not used as an explanatory variable in the present conceptual framing.

### 2.4 Disciplinary and proficiency-based variation in acceptance

Learners in higher education differ in disciplinary background and English proficiency, and these contextual factors may be relevant when examining acceptance of AI-assisted English learning tools. Similar AI-supported affordances may be appraised through different evaluative criteria depending on academic field, language-learning demands, and baseline linguistic capacity (Becher and Trowler, 2001; Scherer et al., 2019). In this study, disciplinary affiliation and English proficiency are used as contextual grouping variables for examining mean-level variation and descriptive subgroup-specific association patterns.

Disciplinary background may be relevant because students from different academic fields encounter different task formats, communicative expectations, standards of evidence, and requirements for language use (Becher and Trowler, 2001; Fry and Talja, 2007). As a grouping variable, disciplinary affiliation provides a contextual basis for describing association patterns estimated separately within each disciplinary subgroup. This framing does not assume that disciplinary membership causes differential outcomes; rather, it supports exploratory and descriptive reporting of association patterns estimated separately within each disciplinary subgroup.

English proficiency is used as a proxy indicator of learners’ baseline capacity to handle English-learning demands and engage with AI-mediated feedback and practice opportunities. Learners with different proficiency levels may differ in how they interpret feedback, revise language output, manage task complexity, or evaluate the usefulness of AI-supported assistance. As a grouping variable, proficiency serves a contextual purpose by allowing association patterns to be described within cohorts characterized by differing levels of baseline task capacity.

The subgroup analyses in this study are exploratory and descriptive rather than confirmatory. Disciplinary affiliation and English proficiency are used to describe association patterns estimated separately within each subgroup, rather than to test formal between-group differences in regression coefficients or to advance explanatory claims about subgroup-specific processes.

### 2.5 Conceptual summary and analytical framing

The conceptual framing of this study distinguishes baseline acceptance-related constructs from learner psychological resources, affective and evaluative correlates, and contextual grouping variables. PU, PEOU, BI, and satisfaction constitute the baseline acceptance-related constructs. PU and PEOU capture learners’ belief-level appraisals of usefulness and ease of use. BI captures intention to continue using AI-assisted tools, whereas satisfaction captures post-use evaluation of the AI-supported learning experience.

Learning motivation and self-efficacy are examined as learner psychological resources. Motivation represents the value-oriented dimension of AI-assisted English learning and may relate to whether learners perceive AI-supported activities as useful and worthwhile. Self-efficacy represents the capability-related dimension and may relate to whether learners experience AI-supported tasks as manageable. Anxiety and risk perception are examined as affective and evaluative correlates whose direction and salience remain empirical questions. Disciplinary affiliation and English proficiency are used as contextual grouping variables for examining mean-level variation and descriptive subgroup-specific association patterns.

Because this study employs a cross-sectional survey design, the framework is used to organize regression-based association analyses, mean-level group comparisons, and exploratory subgroup-specific descriptive analyses. It does not imply causal ordering, mediation, temporal sequencing, or process-based effects.

## 3 Methodology

### 3.1 Research context and participants

This study adopted a quantitative, cross-sectional survey design to examine psychological factors associated with university students’ acceptance of AI-assisted English language learning tools. The research was conducted at one public comprehensive university in Tianjin, northern China. This setting was appropriate for the present investigation because AI-assisted tools, including automated feedback systems, conversational support tools, writing assistance applications, adaptive practice platforms, and personalized learning resources, had become increasingly common in university-level English learning contexts.

Participants were non-English-major second-year undergraduates enrolled in College English courses. This cohort was selected because second-year students had generally completed the initial transition into university-level English learning and had already taken the College English Test Band 4 (CET-4). Their prior CET-4 results provided a standardized basis for classifying English proficiency, making this group appropriate for examining acceptance of AI-assisted English learning tools across proficiency levels. Participants were recruited from intact College English classes using a convenience cluster sampling strategy.

The inclusion criteria were as follows: participants had to be non-English-major second-year undergraduates enrolled in College English courses at the participating university, have available CET-4 scores for proficiency classification, report current or prior experience using AI-assisted tools for English learning, and provide electronic informed consent before completing the questionnaire. These criteria were adopted to ensure that participants were drawn from a comparable university English learning context, had a standardized basis for English proficiency grouping, and had actual exposure to AI-assisted English learning tools. Responses were excluded if participants did not meet the inclusion criteria, submitted incomplete questionnaires, or failed the attention-check or basic quality-control items.

Data were collected through an online questionnaire. After excluding incomplete responses and responses failing basic quality checks, the final analytical sample consisted of 210 students. All valid respondents reported either current or prior experience using AI-assisted tools for English learning, ensuring that their responses reflected actual exposure rather than purely hypothetical judgments.

Table 1 summarizes the demographic and academic characteristics of the analytical sample, including subgroup sample sizes for gender, academic discipline (Humanities vs. STEM), and CET-4-based English proficiency status. For subgroup analyses, students were classified by academic discipline into Humanities and STEM groups and stratified by English proficiency into Low (<425), Intermediate (425–499), and High (≥500) proficiency levels based on CET-4 score thresholds.

**TABLE 1:** Demographic and academic characteristics of the analytical sample.

Characteristic	Category	*n*	%
Gender	Male	89	42.4
	Female	121	57.6
Academic discipline	Humanities	119	56.7
	STEM	91	43.3
CET-4 proficiency level	Low (<425)	77	36.7
	Intermediate (425–499)	103	49.0
	High (≥500)	30	14.3

Percentages are based on the analytical sample (*N* = 210). Participants were non-English-major second-year undergraduates enrolled in College English courses and were recruited from intact classes at one public comprehensive university in Tianjin, northern China. CET-4, College English Test Band 4. CET-4 scores range from 0 to 710, with 425 as the passing score.

### 3.2 Data collection procedure

Data were collected during the Spring semester of 2025 using the Wenjuanxing online survey platform. The questionnaire was distributed electronically to students enrolled in intact College English classes at the participating university. The study was reviewed and determined to be exempt from formal ethics approval by the Academic Research Office of the School of Foreign Languages at the participating university, as it involved an anonymous, non-interventional online survey in the field of social sciences, collected no personally identifiable information, and presented no more than minimal risk to participants.

Participation was anonymous and voluntary. Before accessing the questionnaire, respondents were presented with an electronic informed consent statement describing the study purpose, estimated completion time, confidentiality and anonymity safeguards, and their right to withdraw at any time without penalty. Only respondents who provided explicit electronic consent were allowed to proceed to the questionnaire.

During data screening, responses were first checked against the inclusion criteria specified in section “3.1 Research context and participants.” Respondents were retained only if they provided electronic informed consent, were enrolled in the target College English cohort, had available CET-4 information for proficiency classification, and reported current or prior experience using AI-assisted tools for English learning. The questionnaire also collected information on students’ frequency of AI-assisted English learning tool use, which was used as a background covariate in the regression models. Incomplete submissions and responses failing the attention-check or basic quality-control items were then excluded from subsequent analyses. After screening, the final analytical sample consisted of 210 students.

### 3.3 Measures and scale adaptation

The measurement instruments used in this study were adapted from established scales and contextualized for AI-assisted English language learning. Unless otherwise noted, all scale items were rated on a five-point Likert scale, with 1 = strongly disagree and 5 = strongly agree. The full adapted instrument, including construct sources, adaptation focus, item codes, and survey items, is provided in Supplementary Appendix A.

The questionnaire was administered in Chinese. The Chinese item wording was developed by contextually adapting established measures to the AI-assisted English learning context and was reviewed by the author team for clarity, semantic consistency, contextual appropriateness, and construct fidelity. The author team comprised university EFL teachers and researchers with bilingual proficiency and specialized expertise in English language education and AI-assisted language learning. The adaptation procedure involved an internal author-team linguistic and conceptual review based on the authors’ bilingual and domain expertise, rather than a formal back-translation protocol or external expert-panel review. The English item wording shown in Supplementary Appendix A is provided for manuscript presentation purposes. Contextual adaptation mainly involved replacing the original technology-, system-, or computer-related referents with wording such as “AI tools” or “AI-assisted English learning,” while retaining the central meaning of the original constructs. For example, perceived usefulness retained its original focus on whether the use of a system is perceived to enhance performance, with the referent shifted to AI tools in the English learning context.

Technology acceptance variables included perceived usefulness (PU), perceived ease of use (PEOU), behavioral intention (BI), and satisfaction. Perceived usefulness was measured using three items contextually adapted from Davis (1989) to capture students’ perceptions of the usefulness of AI tools for English learning efficiency, resource access, and learning support. A representative item is: “Using AI tools helps me improve the efficiency of completing my English learning tasks.” Perceived ease of use was measured using three items adapted from Davis (1989) to assess the perceived ease of learning, adapting to, and using AI tools for English learning. A representative item is: “Learning how to use these AI tools is easy for me.” Behavioral intention was measured using two items adapted from Venkatesh et al. (2012) and was operationalized as students’ positive future-oriented intention toward AI-assisted English learning, including continued use and willingness to recommend AI tools to peers. A representative item is: “I intend to continue using AI tools for learning English in the future.” Satisfaction was measured using five items adapted from Bhattacherjee (2001), reflecting students’ overall post-use evaluation of AI-assisted English learning. A representative item is: “Overall, I am satisfied with my experience of using AI for English learning.”

Learner-related psychological variables included self-efficacy (SE), learning motivation (LM), anxiety (ANX), and risk perception (RP). Self-efficacy was measured using five items adapted from the Computer Self-Efficacy scale (Compeau and Higgins, 1995). The items were contextualized to capture students’ perceived capability to use AI tools to complete and manage English learning tasks, rather than general computer competence. A representative item is: “I know how to use AI tools to complete different types of English learning tasks.” Learning motivation was measured using six items adapted with reference to the Academic Motivation Scale (Vallerand et al., 1992, 1993). In this study, learning motivation was treated as a broad overall motivational orientation toward AI-assisted English learning, rather than as separate motivation regulation subtypes. A representative item is: “Using AI tools increases my interest in learning English.”

Anxiety and risk perception were included to capture different types of potentially negative responses to AI-assisted English learning. Anxiety was measured using six items adapted from Wang and Wang (2019) and was operationalized as an affective response, including unease, worry, or reduced confidence when using AI tools for English learning. A representative item is: “Using AI for English learning makes me feel less confident about my English abilities.” Risk perception was measured using four items adapted from Featherman and Pavlou (2003) and was operationalized as a cognitive appraisal of possible adverse consequences, such as inaccurate AI-generated content, weakened language skills, reduced independent thinking, and privacy or data risks. A representative item is: “I am concerned that AI-generated content may be inaccurate or misleading.” This distinction clarifies the theoretical boundary between the two constructs: anxiety refers primarily to affective unease associated with AI-assisted learning, whereas risk perception refers to students’ cognitive evaluation of possible negative consequences.

Scale composite scores were computed as the mean of the item responses for each construct. Internal consistency was assessed using Cronbach’s alpha. Composite reliability (CR) and average variance extracted (AVE) are reported in Table 2 as supplementary descriptive indices. These indices were used to describe the reliability and aspects of convergent validity of the adapted scales in the present sample. Constructs with comparatively weaker reliability or AVE values were interpreted with appropriate caution in the subsequent analyses.

**TABLE 2:** Reliability and convergent validity evidence for the study constructs.

Construct	Items	Cronbach’s α	CR	AVE
Perceived usefulness (PU)	3	0.906	0.905	0.761
Perceived ease of use (PEOU)	3	0.921	0.922	0.798
Behavioral intention (BI)	2	0.842	0.845	0.731
Satisfaction	5	0.933	0.933	0.735
Self-efficacy	5	0.922	0.923	0.707
Learning motivation	6	0.929	0.931	0.693
Anxiety	6	0.890	0.895	0.593
Risk perception	4	0.613	0.675	0.363

CR, composite reliability; AVE, average variance extracted.

### 3.4 Data analysis strategy

All statistical analyses were conducted using IBM SPSS Statistics 27.0 (IBM Corp., Armonk, NY, United States) and Stata 18.0 (StataCorp LLC, College Station, TX, United States). Categorical sample characteristics were summarized using frequencies and percentages (Table 1). The main study variables were summarized using means, standard deviations, minimum values, and maximum values (Table 3). Scale composite scores were calculated as item-average scores for each construct so that all construct-level variables were retained on the original five-point response scale.

**TABLE 3:** Descriptive statistics of the main study variables.

Variable	*N*	*M*	SD	Min	Max
Perceived usefulness (PU)	210	4.048	0.671	1	5
Perceived ease of use (PEOU)	210	3.984	0.707	1	5
Behavioral intention (BI)	210	4.000	0.714	1	5
Satisfaction	210	3.932	0.675	1	5
Self-efficacy	210	3.810	0.680	1	5
Learning motivation	210	3.810	0.676	1	5
Anxiety	210	3.868	0.673	1	5
Risk perception	210	3.686	0.625	1	5

M, mean; SD, standard deviation. All variables were calculated as item-average scores on a five-point scale.

Internal consistency and convergent validity were examined using Cronbach’s alpha, composite reliability (CR), and average variance extracted (AVE) (Table 2). Pearson correlations among the main study variables, together with the square roots of AVE, were calculated as supplementary correlation and validity information (Supplementary Table 4).

To examine associations between learner-related psychological variables and acceptance outcomes, full-sample adjusted ordinary least squares (OLS) linear regression models were estimated. The dependent variables were perceived usefulness (PU), perceived ease of use (PEOU), behavioral intention (BI), and satisfaction. Self-efficacy, learning motivation, anxiety, and risk perception were examined as focal psychological correlates in separate adjusted models (i.e., not entered simultaneously). Each model adjusted for gender, academic discipline, CET-4 score, and frequency of AI-assisted English learning tool use. Categorical covariates were dummy-coded as appropriate. The full-sample regression coefficients and selected model-level indices are reported in Table 4, with additional coefficient estimates and model summaries provided in Supplementary Tables 1a,b.

**TABLE 4:** Full-sample adjusted regression models for acceptance outcomes.

Outcome	Focal predictor	B	SE	β	95% CI for B	*P*	R^2^	Adj. R^2^	Cohen’s f^2^	*N*
PU	Self-efficacy	0.363	0.037	0.614	[0.289, 0.437]	<0.001	0.442	0.426	0.794	210
	Learning motivation	0.310	0.029	0.625	[0.252, 0.368]	<0.001	0.444	0.428	0.799	210
	Risk perception	0.383	0.062	0.476	[0.260, 0.506]	<0.001	0.298	0.278	0.426	210
	Anxiety	0.291	0.037	0.584	[0.219, 0.364]	<0.001	0.394	0.376	0.650	210
PEOU	Self-efficacy	0.412	0.033	0.661	[0.348, 0.477]	<0.001	0.510	0.496	1.042	210
	Learning motivation	0.350	0.028	0.669	[0.295, 0.405]	<0.001	0.506	0.492	1.028	210
	Risk perception	0.401	0.065	0.472	[0.272, 0.529]	<0.001	0.306	0.286	0.442	210
	Anxiety	0.281	0.039	0.535	[0.204, 0.359]	<0.001	0.356	0.337	0.553	210
BI	Self-efficacy	0.254	0.029	0.605	[0.197, 0.311]	<0.001	0.455	0.439	0.835	210
	Learning motivation	0.226	0.019	0.643	[0.188, 0.264]	<0.001	0.487	0.472	0.950	210
	Risk perception	0.238	0.044	0.417	[0.152, 0.324]	<0.001	0.272	0.251	0.374	210
	Anxiety	0.227	0.024	0.640	[0.179, 0.274]	<0.001	0.478	0.463	0.917	210
Satisfaction	Self-efficacy	0.781	0.043	0.787	[0.700, 0.866]	<0.001	0.666	0.656	1.995	210
	Learning motivation	0.674	0.035	0.810	[0.605, 0.743]	<0.001	0.680	0.671	2.131	210
	Risk perception	0.566	0.105	0.419	[0.359, 0.773]	<0.001	0.247	0.225	0.328	210
	Anxiety	0.451	0.062	0.539	[0.328, 0.574]	<0.001	0.344	0.325	0.524	210

Each row represents a separate adjusted linear regression model. All models were adjusted for gender, academic discipline, CET-4 score, and frequency of AI-assisted English learning tool use. B, unstandardized coefficient; β, standardized coefficient; CI, confidence interval; SE, standard error. SEs, *p*-values, and 95% CIs were based on robust standard errors. PU, perceived usefulness; PEOU, perceived ease of use; BI, behavioral intention.

Regression diagnostics were examined to support transparent interpretation of the full-sample adjusted models. Multicollinearity was evaluated using variance inflation factors (VIFs) and tolerance values. Residual normality was examined using Q-Q plots and the Shapiro-Wilk test. Heteroscedasticity was assessed using White tests, and functional-form adequacy was examined using RESET tests. Outliers and potentially influential observations were inspected using Cook’s distance, leverage values, and studentized residuals. Because heteroscedasticity was detected in Models 1, 2, and 3, robust standard errors were used for coefficient inference in all full-sample adjusted models. The multicollinearity, residual, heteroscedasticity, functional-form, and influence diagnostics are reported in Supplementary Tables 2, 3a–c. These diagnostic checks were used to support transparent and cautious interpretation of the regression estimates rather than to support causal or mechanistic claims.

Mean-level differences in acceptance outcomes were examined by academic discipline and CET-4-based English proficiency level. Levene’s test was used to assess the homogeneity of variance assumption. When the assumption was met, one-way ANOVA was used; when the assumption was not met, Welch’s test was used. Eta squared (η^2^) was reported as an effect-size estimate for the group comparisons. These mean-level group comparison results are reported in Table 5. For the three-level English proficiency grouping, Tukey HSD *post hoc* comparisons were conducted to examine pairwise mean differences among the Low, Intermediate, and High proficiency groups. These *post hoc* comparisons are reported in Supplementary Table 5. In cases where *post hoc* comparisons were reported following a non-significant omnibus test, they were treated as supplementary descriptive information only.

**TABLE 5:** Mean-level differences in acceptance outcomes by academic discipline and English proficiency.

Outcome	Grouping variable	Group means, M ± SD	Levene *p*	Test used	F/Welch F	df	*P*	η^2^
PU	Academic discipline	STEM: 3.91 ± 0.72; Humanities: 4.15 ± 0.62	0.709	ANOVA	7.12	1, 208	0.008	0.033
PEOU	Academic discipline	STEM: 3.85 ± 0.78; Humanities: 4.09 ± 0.63	0.037	Welch	5.91	1, 169.995	0.016	0.029
BI	Academic discipline	STEM: 3.86 ± 0.73; Humanities: 4.11 ± 0.69	0.880	ANOVA	6.09	1, 208	0.014	0.028
Satisfaction	Academic discipline	STEM: 3.84 ± 0.69; Humanities: 4.00 ± 0.66	0.395	ANOVA	2.78	1, 208	0.097	0.013
PU	English proficiency	Low: 4.10 ± 0.68; Intermediate: 4.10 ± 0.61; High: 3.72 ± 0.78	0.472	ANOVA	4.25	2, 207	0.016	0.039
PEOU	English proficiency	Low: 4.09 ± 0.70; Intermediate: 4.02 ± 0.64; High: 3.60 ± 0.85	0.172	ANOVA	5.59	2, 207	0.004	0.051
BI	English proficiency	Low: 4.02 ± 0.73; Intermediate: 4.04 ± 0.69; High: 3.80 ± 0.74	0.739	ANOVA	1.40	2, 207	0.248	0.013
Satisfaction	English proficiency	Low: 3.95 ± 0.65; Intermediate: 4.01 ± 0.66; High: 3.63 ± 0.72	0.806	ANOVA	3.85	2, 207	0.023	0.036

Group means and standard deviations are reported as item-average scores on a five-point scale. Welch’s test was used when the homogeneity of variance assumption was not met. η^2^, eta squared. For academic discipline, STEM *n* = 91 and humanities *n* = 119; for English proficiency, low *n* = 77, intermediate *n* = 103, and high *n* = 30. PU, perceived usefulness; PEOU, perceived ease of use; BI, behavioral intention. *Post hoc* comparisons for English proficiency groups are reported in Supplementary Table 5.

Exploratory subgroup-specific adjusted regression models were conducted by academic discipline and CET-4-based English proficiency level to describe whether association patterns appeared broadly similar across learner groups. Unlike the full-sample adjusted models, the subgroup-specific regression models used conventional OLS standard errors and were reported only as descriptive supplementary evidence. Discipline-specific coefficient estimates and model summaries are reported in Supplementary Tables 6a,b. Proficiency-specific coefficient estimates and model summaries are reported in Supplementary Tables 7a,b. These subgroup-specific models were interpreted cautiously because subgroup sizes were unequal and the High-proficiency subgroup was relatively small. No group-by-correlate interaction tests, formal tests of between-group differences in regression coefficients, or confirmatory moderation analyses were conducted. Accordingly, subgroup-specific findings were treated as exploratory and descriptive patterns rather than as evidence that regression coefficients differed across groups.

Given the cross-sectional survey design, all regression and group-comparison findings were interpreted as associative and descriptive rather than causal. The analyses were not designed to establish temporal ordering, causal effects, or underlying explanatory processes in students’ acceptance of AI-assisted English learning tools.

## 4 Results

The results are presented in four sections. Section “4.1 Descriptive statistics and measurement evidence” reports descriptive statistics and measurement evidence for the analytical sample and main study variables. Section “4.2 Full-sample adjusted regression models: associations between psychological correlates and acceptance outcomes” addresses RQ1 by presenting full-sample adjusted regression models examining associations between learner-related psychological correlates and acceptance outcomes. Section “4.3 Mean-level differences in acceptance outcomes by academic discipline and English proficiency” reports mean-level differences in acceptance outcomes by academic discipline and English proficiency. Section “4.4 Exploratory subgroup-specific regression patterns” presents exploratory subgroup-specific regression patterns. Consistent with the cross-sectional, exploratory design of this study and the limits of inference related to unobserved confounding, possible bidirectional associations, and measurement error, the regression findings are interpreted strictly as descriptive and associative patterns rather than as causal relationships.

### 4.1 Descriptive statistics and measurement evidence

The analytical sample consisted of 210 non-English-major second-year undergraduates enrolled in College English courses. As shown in Tables 1, 89 participants were male (42.4%) and 121 were female (57.6%). Regarding academic discipline, 119 students were from Humanities disciplines (56.7%) and 91 were from STEM disciplines (43.3%). Based on CET-4 scores, 77 students were classified into the Low-proficiency group (<425; 36.7%), 103 into the Intermediate-proficiency group (425–499; 49.0%), and 30 into the High-proficiency group (≥500; 14.3%).

Table 2 reports reliability and convergent validity evidence for the study constructs. Cronbach’s alpha values ranged from 0.613 to 0.933, composite reliability (CR) values ranged from.675 to.933, and average variance extracted (AVE) values ranged from 0.363 to 0.798. Most constructs showed satisfactory internal consistency and acceptable supplementary convergent validity evidence in the present sample. Risk perception exhibited lower measurement indices (α = 0.613, CR = 0.675, AVE = 0.363), indicating weaker convergent validity evidence. Findings involving this construct should therefore be interpreted with particular caution.

Descriptive statistics for the main study variables are presented in Table 3. The four acceptance outcomes showed generally high mean levels on the five-point scale, with mean scores ranging from 3.932 for satisfaction to 4.048 for perceived usefulness. The learner-related psychological variables also showed mean scores above the scale midpoint, ranging from 3.686 for risk perception to 3.868 for anxiety. As shown in Table 3, the minimum and maximum values were 1 and 5 for all constructs, indicating that observed responses covered the full response range.

Supplementary Table 4 reports Pearson correlations among the main study variables, together with the square roots of AVE. All off-diagonal correlations were statistically significant at *p* < 0.001. The four acceptance outcomes were positively intercorrelated. Self-efficacy, learning motivation, anxiety, and risk perception also showed positive bivariate correlations with the four acceptance outcomes. These correlations are reported as supplementary descriptive information to contextualize the adjusted regression analyses, not as standalone evidence of causal relationships.

### 4.2 Full-sample adjusted regression models: associations between psychological correlates and acceptance outcomes

To address RQ1, full-sample adjusted OLS regression models were estimated to examine associations between learner-related psychological correlates and acceptance outcomes. In accordance with the final analytical strategy, self-efficacy, learning motivation, risk perception, and anxiety were examined as focal correlates in separate adjusted models rather than entered simultaneously. Each model adjusted for gender, academic discipline, CET-4 score, and frequency of AI-assisted English learning tool use. Robust standard errors were used for coefficient inference in all full-sample adjusted models. The main results are reported in Table 4, with additional coefficient estimates and model summaries provided in Supplementary Tables 1a,b.

For perceived usefulness, all four focal correlates showed statistically significant positive associations in the separate adjusted models (all *p* < 0.001). Standardized coefficients ranged from β = 0.476 for risk perception to β = 0.625 for learning motivation, and adjusted R^2^ values ranged from 0.278 to 0.428 across the four separate models.

For perceived ease of use, self-efficacy, learning motivation, risk perception, and anxiety were also positively associated with the outcome (all *p* < 0.001). Standardized coefficients ranged from β = 0.472 for risk perception to β = 0.669 for learning motivation, and adjusted R^2^ values ranged from 0.286 to 0.496 across the four separate models.

For behavioral intention, all four focal correlates showed statistically significant positive associations (all *p* < 0.001). Standardized coefficients ranged from β = 0.417 for risk perception to β = 0.643 for learning motivation, and adjusted R^2^ values ranged from 0.251 to 0.472 across the four separate models.

For satisfaction, the same general pattern was observed. Self-efficacy, learning motivation, risk perception, and anxiety were each positively associated with satisfaction (all *p* < 0.001). Standardized coefficients ranged from β = 0.419 for risk perception to β = 0.810 for learning motivation, and adjusted R^2^ values ranged from 0.225 to 0.671 across the four separate models. Among the four acceptance outcomes, the models for satisfaction showed the highest adjusted R^2^ values when self-efficacy and learning motivation were examined as focal correlates.

Across the 16 full-sample adjusted models, all models were statistically significant (all *p* < 0.001), with F(6, 203) values ranging from 10.93 to 75.35. Overall, the full-sample adjusted models showed consistent positive associations between the four learner-related psychological correlates and the four acceptance outcomes. Because each focal correlate was examined in a separate adjusted model, these findings should be interpreted as adjusted association patterns for individual psychological variables, not as estimates from a simultaneous model including all psychological variables together.

Supplementary diagnostic checks supported a cautious interpretation of the full-sample regression results. Multicollinearity was not a serious concern, with VIF values ranging from 1.06 to 1.10 and tolerance values ranging from 0.9079 to 0.9448. Although the Shapiro-Wilk test indicated a formal deviation from residual normality (W = 0.9715, *p* < 0.001), this diagnostic limitation was not treated as precluding coefficient reporting given the sample size (*N* = 210). White tests indicated heteroscedasticity in the models for perceived usefulness, perceived ease of use, and behavioral intention, but not in the model for satisfaction; robust standard errors were therefore applied across all full-sample adjusted models. RESET tests indicated acceptable functional form for the first three outcome models, whereas the satisfaction model showed a slight specification concern (*p* = 0.006). Influence diagnostics did not indicate severe influential observations. Taken together, these diagnostics do not indicate severe problems that would preclude reporting the full-sample regression results. Nevertheless, the findings should be interpreted as descriptive and associative patterns.

### 4.3 Mean-level differences in acceptance outcomes by academic discipline and English proficiency

To complement the full-sample adjusted models, mean-level differences in the four acceptance outcomes were examined by academic discipline and English proficiency. Table 5 reports the group means, test statistics, and η^2^ values, and Supplementary Table 5 reports Tukey HSD post hoc comparisons among the English proficiency groups.

For academic discipline, statistically significant mean-level differences were observed for perceived usefulness, perceived ease of use, and behavioral intention. Humanities students reported higher mean levels than STEM students for perceived usefulness (Humanities: 4.15 ± 0.62; STEM: 3.91 ± 0.72), F(1, 208) = 7.12, *p* = 0.008, η^2^ = 0.033. A similar disciplinary difference was observed for perceived ease of use. Because the homogeneity of variance assumption was not met, Welch’s test was used. Humanities students reported a higher mean level of perceived ease of use than STEM students (Humanities: 4.09 ± 0.63; STEM: 3.85 ± 0.78), Welch’s F(1, 169.995) = 5.91, *p* = 0.016, η^2^ = 0.029. Behavioral intention also differed by academic discipline, with Humanities students reporting a higher mean level than STEM students (Humanities: 4.11 ± 0.69; STEM: 3.86 ± 0.73), F(1, 208) = 6.09, *p* = 0.014, η^2^ = 0.028. By contrast, the mean-level difference in satisfaction was not statistically significant, F(1, 208) = 2.78, *p* = 0.097, η^2^ = 0.013. Overall, the η^2^ values for the discipline-based comparisons were small.

For English proficiency, statistically significant mean-level differences were found for perceived usefulness, perceived ease of use, and satisfaction, whereas behavioral intention did not differ significantly across proficiency groups. For perceived usefulness, the omnibus test was statistically significant, F(2, 207) = 4.25, *p* = 0.016, η^2^ = 0.039. The group means were 4.10 ± 0.68 for the Low-proficiency group, 4.10 ± 0.61 for the Intermediate-proficiency group, and 3.72 ± 0.78 for the High-proficiency group. Tukey HSD comparisons indicated that both the Low-proficiency group (mean difference = 0.377, *p* = 0.009) and the Intermediate-proficiency group (mean difference = 0.381, *p* = 0.006) reported higher perceived usefulness than the High-proficiency group. The Low- and Intermediate-proficiency groups did not differ significantly from each other (*p* = 0.968).

For perceived ease of use, mean levels also differed across proficiency groups, F(2, 207) = 5.59, *p* = 0.004, η^2^ = 0.051. The group means were 4.09 ± 0.70 for the Low-proficiency group, 4.02 ± 0.64 for the Intermediate-proficiency group, and 3.60 ± 0.85 for the High-proficiency group. Tukey HSD comparisons showed that both the Low-proficiency group (mean difference = 0.487, *p* = 0.001) and the Intermediate-proficiency group (mean difference = 0.419, *p* = 0.004) reported higher perceived ease of use than the High-proficiency group, whereas the Low- and Intermediate-proficiency groups did not differ significantly from each other (*p* = 0.520).

For behavioral intention, the mean-level difference across proficiency groups was not statistically significant, F(2, 207) = 1.40, *p* = 0.248, η^2^ = 0.013. Although *post hoc* comparisons for behavioral intention are reported in Supplementary Table 5 for completeness, they are reported only as supplementary descriptive information because the omnibus test was not statistically significant.

For satisfaction, mean levels differed across proficiency groups, F(2, 207) = 3.85, *p* = 0.023, η^2^ = 0.036. The group means were 3.95 ± 0.65 for the Low-proficiency group, 4.01 ± 0.66 for the Intermediate-proficiency group, and 3.63 ± 0.72 for the High-proficiency group. Tukey HSD comparisons indicated that both the Low-proficiency group (mean difference = 0.324, *p* = 0.025) and the Intermediate-proficiency group (mean difference = 0.381, *p* = 0.006) reported higher satisfaction than the High-proficiency group, whereas the Low- and Intermediate-proficiency groups did not differ significantly from each other (*p* = 0.570).

Taken together, the mean-level comparisons showed that Humanities students reported higher mean levels of perceived usefulness, perceived ease of use, and behavioral intention than STEM students, whereas satisfaction did not differ significantly by discipline. Proficiency-related mean-level differences were observed for perceived usefulness, perceived ease of use, and satisfaction, with the High-proficiency group reporting lower mean levels than the Low- and Intermediate-proficiency groups on these outcomes. Behavioral intention did not differ significantly across proficiency groups. These mean-level patterns provide descriptive background for the exploratory subgroup-specific regression analyses reported in section “4.4 Exploratory subgroup-specific regression patterns.”

### 4.4 Exploratory subgroup-specific regression patterns

Exploratory subgroup-specific adjusted regression models were estimated to describe within-group association patterns by academic discipline and English proficiency. These models are reported as descriptive supplementary evidence and use conventional OLS standard errors rather than robust standard errors. No group-by-correlate interaction tests, formal between-group coefficient comparison tests, or confirmatory moderation analyses were conducted. Therefore, the subgroup-specific results should be interpreted as within-subgroup association patterns only, not as evidence of moderation or statistically confirmed between-group differences in regression coefficients. Full coefficient estimates and model-level summaries are reported in Supplementary Tables 6a,b for academic discipline and Supplementary Tables 7a,b for English proficiency.

#### 4.4.1 Discipline-specific exploratory patterns

Within the STEM subgroup (*n* = 91), all four focal correlates showed statistically significant positive associations with the four acceptance outcomes in the separate adjusted models. For perceived usefulness, perceived ease of use, behavioral intention, and satisfaction, the coefficients for self-efficacy, learning motivation, risk perception, and anxiety were all positive and statistically significant (all *p* < 0.001). The adjusted R^2^ values across the STEM subgroup models ranged from 0.330 to 0.708. Within this subgroup, the satisfaction models with self-efficacy and learning motivation as focal correlates showed comparatively large adjusted R^2^ values.

Within the Humanities subgroup (*n* = 119), a broadly similar descriptive pattern was observed. Self-efficacy, learning motivation, risk perception, and anxiety were positively associated with perceived usefulness, perceived ease of use, behavioral intention, and satisfaction in the separate adjusted models. Most coefficients were statistically significant at *p* < 0.001; the association between risk perception and satisfaction was also statistically significant, B = 0.359, SE = 0.126, β = 0.249, *p* = 0.005, 95% CI [0.109, 0.609]. The adjusted R^2^ values across the Humanities subgroup models ranged from 0.097 to 0.636. Within this subgroup, the satisfaction models with self-efficacy and learning motivation as focal correlates also showed comparatively large adjusted R^2^ values.

Taken together, the discipline-specific models showed positive within-subgroup association patterns in both STEM and Humanities students. However, these patterns are descriptive only. Differences in coefficient sizes, adjusted R^2^ values, or statistical significance across the two disciplinary subgroups should not be interpreted as formal between-group differences because no interaction or coefficient-comparison tests were conducted.

#### 4.4.2 Proficiency-specific exploratory patterns

Within the Low-proficiency subgroup (*n* = 77), the subgroup-specific models showed generally positive association patterns between the focal correlates and acceptance outcomes. Self-efficacy, learning motivation, and anxiety were positively and statistically associated with perceived usefulness, perceived ease of use, behavioral intention, and satisfaction in the separate adjusted models. Risk perception was positively and statistically associated with perceived usefulness, perceived ease of use, and behavioral intention. However, risk perception was not statistically associated with satisfaction at the coefficient level, B = 0.286, SE = 0.146, β = 0.228, *p* = 0.054, 95% CI [−0.005, 0.576]. The corresponding model-level test was statistically significant, F(6, 70) = 2.43, *p* = 0.034.

Within the Intermediate-proficiency subgroup (*n* = 103), all four focal correlates showed statistically significant positive associations with the four acceptance outcomes in the separate adjusted models (all *p* < 0.001). This pattern was observed for perceived usefulness, perceived ease of use, behavioral intention, and satisfaction. The satisfaction models with self-efficacy and learning motivation as focal correlates showed comparatively large adjusted R^2^ values within this subgroup.

Within the High-proficiency subgroup (*n* = 30), the subgroup-specific models also showed positive and statistically significant associations between the focal correlates and the acceptance outcomes in the separate adjusted models. However, because this subgroup was small, the coefficient estimates may be less stable and should be interpreted with particular caution. The High-proficiency subgroup results are reported to describe within-group association patterns and should not be used to make confirmatory claims about proficiency-related differences in regression coefficients.

Overall, the proficiency-specific models showed broadly positive within-subgroup association patterns across the Low-, Intermediate-, and High-proficiency groups. The main exception was the risk perception coefficient in the Low-proficiency satisfaction model, which did not reach coefficient-level statistical significance, B = 0.286, SE = 0.146, β = 0.228, *p* = 0.054, 95% CI [−0.005, 0.576]. Therefore, this coefficient is not treated as a significant focal-correlate finding, even though the corresponding model-level test was statistically significant. These subgroup-specific findings are exploratory and descriptive. They should not be interpreted as moderation findings or as evidence that the psychological correlates differed statistically across proficiency groups.

## 5 Discussion

### 5.1 Summary of principal findings

This study examined university students’ acceptance of AI-assisted English language learning tools by integrating TAM-based acceptance outcomes with learner-related psychological, affective, and evaluative correlates. Within an associational and exploratory framework, three main findings warrant discussion. First, in the full analytical sample, learning motivation, self-efficacy, anxiety, and risk perception were each positively associated, in separate adjusted regression models, with perceived usefulness, perceived ease of use, behavioral intention, and satisfaction. This pattern should not be interpreted as implying that anxiety or risk perception function as inherently beneficial factors or positive resources. Rather, the findings indicate that students’ acceptance-related evaluations covaried with learner-centered psychological and evaluative measures in this sample, without implying temporal ordering, causal direction, or underlying mechanisms.

Second, the results were broadly consistent with the theoretically informed expectations for learning motivation and self-efficacy. Learning motivation was positively associated with all four acceptance outcomes, including perceived usefulness, satisfaction, and behavioral intention. Self-efficacy was also positively associated with all four outcomes, including perceived ease of use and satisfaction. These patterns are consistent with the view that students who report stronger learning-related value appraisals and greater perceived capability in managing AI-supported tasks may evaluate AI-assisted English learning tools more favorably.

Third, the findings for anxiety, risk perception, academic discipline, and English proficiency require a more cautious interpretation. Anxiety and risk perception were treated as exploratory correlates, and their positive associations with acceptance outcomes should not be read as evidence that anxiety or perceived risk is desirable or facilitative in AI-assisted learning. Rather, these results may suggest that affective tension, risk awareness, and favorable acceptance evaluations can coexist in AI-assisted English learning contexts. In addition, mean-level group differences and subgroup-specific regression patterns should be kept analytically distinct. The former describe differences in average acceptance outcomes across discipline and proficiency groups, whereas the latter describe within-subgroup association patterns. Because no interaction tests or formal coefficient-comparison tests were conducted, the subgroup-specific results do not constitute formal evidence of between-group differences in regression coefficients.

### 5.2 Motivation and self-efficacy as theoretically expected correlates

The full-sample adjusted regression models showed consistent positive associations between learning motivation and the four acceptance outcomes. This finding is consistent with Self-Determination Theory, which emphasizes the importance of perceived value, engagement, and goal relevance in learning. In the present context, students with stronger motivation for English learning may be more likely to perceive AI-assisted tools as relevant to their language learning goals. AI-supported feedback, practice opportunities, revision assistance, and personalized guidance may be evaluated more favorably when learners already perceive English learning as meaningful and worth sustained effort. This interpretation is particularly relevant to the positive associations between learning motivation and perceived usefulness, behavioral intention, and satisfaction.

Self-efficacy also showed consistent positive associations with perceived usefulness, perceived ease of use, behavioral intention, and satisfaction. This pattern is consistent with Social Cognitive Theory and with technology acceptance research that links perceived capability to favorable technology-related evaluations. In AI-assisted English learning, self-efficacy may be especially relevant because students are not simply operating a digital interface. They are also actively interpreting automated feedback, judging the quality of AI-generated suggestions, revising language output, and deciding how to incorporate automated support into their own learning process. These complex tasks may require a degree of metacognitive monitoring and evaluation, including the ability to critically appraise whether an AI-generated response is contextually appropriate, grammatically accurate, and compatible with one’s intended meaning. Students with stronger self-efficacy may therefore experience this interactive process as more manageable, which is consistent with the observed association between self-efficacy and perceived ease of use. The positive association between self-efficacy and satisfaction also appears plausible because students who feel more capable of managing AI-supported learning tasks may evaluate the overall learning experience more positively.

These findings should nevertheless be interpreted as concurrent associations rather than causal relationships. The cross-sectional design does not establish temporal ordering, and each psychological variable was examined as a focal correlate in a separate adjusted model rather than as part of a simultaneous model including all psychological variables together. Therefore, the results do not show that motivation or self-efficacy independently produces higher acceptance outcomes. They indicate that, in this sample, students reporting stronger motivation or self-efficacy also tended to report more favorable evaluations of AI-assisted English learning tools. This distinction is important because favorable experiences with AI-assisted learning may also be associated with stronger motivation and self-efficacy over time, suggesting a potentially reciprocal relationship that longitudinal designs should examine.

### 5.3 Anxiety and risk perception as exploratory, context-sensitive correlates

Anxiety was positively associated with all four acceptance outcomes in the full-sample adjusted models. This finding may appear counterintuitive if anxiety is assumed to operate only as a universal barrier to technology acceptance. However, the present study did not formulate a directional expectation for anxiety, and the result should be interpreted as an exploratory concurrent association. In AI-assisted English learning, anxiety may arise when students are highly attentive to their performance, aware of gaps in their English output, or concerned about the quality of their interaction with AI-generated feedback. Such anxiety may coexist with favorable perceptions of usefulness or intention when students also view the tool as a relevant instrument to address their learning needs.

A Control-Value Theory perspective provides a cautious way to interpret this pattern. Affective anxiety is often associated with both high perceived task value and subjective uncertainty about performance outcomes. Students who care deeply about English learning and engage seriously with AI-supported tasks may report some anxiety while simultaneously recognizing the potential utility of AI-assisted feedback, revision, and practice to improve their work. This does not mean that anxiety is pedagogically desirable or that it actively improves acceptance. A more appropriate interpretation is that anxiety, in this sample, did not correspond to lower acceptance evaluations in the separate adjusted models. The finding therefore cautions against treating anxiety as a uniformly negative correlate across all AI-assisted language learning contexts.

Risk perception also showed positive associations with acceptance outcomes in the full sample, but this result should be interpreted with particular caution. First, risk perception was treated as an exploratory correlate. Second, the measurement evidence for this construct was weaker than that for the other constructs, with lower convergent validity evidence than the main acceptance and psychological-resource measures (AVE = 0.363, below conventional thresholds). This limitation may have affected the precision and stability of its estimated associations. Third, the cross-sectional design does not indicate whether risk awareness preceded, followed, or co-occurred with favorable acceptance evaluations. One possible interpretation is that students who are more deeply engaged with AI-assisted learning may also become more aware of privacy, reliability, dependence, or academic-integrity concerns. Another possibility is that students can recognize potential risks through a form of pragmatic calculus while still perceiving AI-assisted English learning tools as useful and manageable. These interpretations remain tentative.

Taken together, the findings for anxiety and risk perception suggest that affective and evaluative responses to AI-assisted English learning should not be reduced to simple positive or negative categories. Students may evaluate AI tools favorably while also experiencing uncertainty, concern, or risk awareness. For this reason, anxiety and risk perception are best discussed as context-sensitive correlates rather than as stable barriers or positive resources.

### 5.4 Mean-level differences by academic discipline and English proficiency

The mean-level comparisons showed that Humanities students reported higher mean levels of perceived usefulness, perceived ease of use, and behavioral intention than STEM students, whereas satisfaction did not differ significantly by discipline. The effect sizes for the significant discipline-based comparisons were small. These findings may suggest that disciplinary background is related to how students evaluate AI-assisted English learning tools at the descriptive mean level. However, they should not be interpreted as indicating that academic discipline itself explains or produces differences in acceptance.

One possible interpretation is that Humanities students may perceive AI-assisted English learning tools as more closely aligned with text-based, communicative, and revision-oriented learning activities. AI-supported functions such as explanation, paraphrasing, drafting, feedback, and language revision may appear especially relevant to learning tasks that emphasize expression and interpretation. STEM students may also value AI-assisted English learning, but they may evaluate such tools through somewhat different task criteria, such as efficiency, precision, or direct relevance to specific technical communication needs. Because these disciplinary orientations were not directly measured in the present study, this interpretation should remain tentative. These mean-level observations do not indicate that disciplinary background causes differential acceptance. Rather, they describe how average scores varied across groups in this sample.

The proficiency-based mean-level comparisons showed significant differences for perceived usefulness, perceived ease of use, and satisfaction, but not for behavioral intention. Low- and Intermediate-proficiency students reported higher mean levels than High-proficiency students for perceived usefulness, perceived ease of use, and satisfaction. This pattern may suggest that students with lower or intermediate English proficiency perceive greater practical value in AI-assisted English learning tools, possibly because such tools provide accessible scaffolding, immediate feedback, and revision support that accommodates their current language capacity. High-proficiency students may evaluate AI-assisted tools more critically, perceive less added value over their existing skills, or require more advanced forms of support to feel equally satisfied.

The absence of a significant proficiency-related mean difference in behavioral intention is also meaningful. Although High-proficiency students reported lower mean levels of perceived usefulness, perceived ease of use, and satisfaction, their intention to use AI-assisted English learning tools did not differ significantly from that of the other proficiency groups. This may suggest that continued willingness to use AI tools can remain relatively similar across proficiency groups even when evaluative judgments differ, potentially due to generalized academic utility. However, this interpretation should be treated cautiously because the High-proficiency subgroup was relatively small. As with the discipline-based findings, these proficiency-based differences describe mean-level variation rather than causal effects of proficiency on acceptance.

### 5.5 Exploratory subgroup-specific association patterns

The subgroup-specific regression analyses were conducted to describe within-group association patterns by academic discipline and English proficiency. These analyses should be interpreted as exploratory and descriptive only. They used ordinary OLS standard errors rather than robust standard errors, and no group-by-correlate interaction tests, formal coefficient-comparison tests, or confirmatory moderation analyses were conducted. Therefore, differences in coefficient size, statistical significance, or model fit across subgroups should not be interpreted as evidence that the associations differed statistically between groups.

Within the academic discipline subgroups, positive associations between the focal psychological correlates and acceptance outcomes were observed in both STEM and Humanities students. In the STEM subgroup, self-efficacy, learning motivation, risk perception, and anxiety were positively associated with perceived usefulness, perceived ease of use, behavioral intention, and satisfaction in the separate adjusted models. In the Humanities subgroup, a broadly similar descriptive pattern was observed. These findings suggest that the positive full-sample association patterns were not confined to one disciplinary group. However, because no formal tests of between-discipline coefficient differences were conducted, the results should be described as parallel within-subgroup patterns rather than as evidence of discipline-specific association strength.

The proficiency-specific subgroup analyses also showed broadly positive within-subgroup association patterns. In the Low-proficiency subgroup (*n* = 77), self-efficacy, learning motivation, and anxiety were positively associated with all four acceptance outcomes in the separate adjusted models. Risk perception was positively associated with perceived usefulness, perceived ease of use, and behavioral intention. However, risk perception was not statistically associated with satisfaction at the coefficient level, because its *p*-value was slightly above the conventional 0.05 threshold (*p* = 0.054) and the confidence interval crossed zero [−0.005, 0.576]. This result should not be treated as a significant focal-correlate finding, even though the corresponding model-level test was statistically significant, F(6, 70) = 2.43, *p* = 0.034. This distinction is important because global model-level significance does not mean that every individual focal coefficient in the model is estimated with sufficient precision.

In the Intermediate-proficiency subgroup, all four focal correlates were positively associated with all four acceptance outcomes in the separate adjusted models. In the High-proficiency subgroup, the subgroup-specific models also showed positive associations. However, the High-proficiency subgroup contained only 30 students, which limits the stability and precision of the coefficient estimates and increases their sensitivity to sampling variation. Therefore, the High-proficiency subgroup results should be interpreted with particular caution and used only to describe the observed within-group patterns in this sample rather than generalizable subpopulation behaviors.

Overall, the subgroup-specific findings may suggest that the positive associations observed in the full sample were broadly reflected across discipline and proficiency groups. However, they do not show that academic discipline or English proficiency statistically moderated or changed the strength of the associations. Future studies with larger subgroup samples and formal multi-group designs would be needed to examine whether these descriptive patterns are stable across learner groups.

### 5.6 Practical implications for AI-assisted English learning

The findings offer several practical implications for AI-assisted English learning in higher education, provided that these implications are interpreted as pedagogical considerations derived from an associational study rather than as tested instructional interventions. Because the present study was cross-sectional and did not evaluate specific instructional interventions, the following implications are intended to support local pedagogical reflection and context-sensitive implementation rather than to prescribe universal teaching or institutional models.

For language teachers, the recurring positive associations involving learning motivation and self-efficacy suggest that AI-assisted English learning activities may be more meaningful when they are connected to concrete learning goals and manageable task procedures. Teachers may therefore consider embedding AI tools within communicative and language-focused tasks, such as revising written drafts, expanding vocabulary choices, improving expression, practicing dialogue, receiving formative feedback, or comparing alternative phrasings. Guided onboarding may also be valuable when students view AI tools primarily as sources of answers rather than as supports for learning. Such support may include prompt-writing demonstrations, feedback interpretation activities, error-detection tasks, and classroom discussions on when AI-generated suggestions should be accepted, revised, or rejected. These practices may help students use AI tools with greater confidence while maintaining active judgment over their own language production.

For learners, the findings point to the importance of strategic and critical AI use. Students may benefit from treating AI tools as learning support systems rather than substitutes for personal effort, authorship, or linguistic judgment. For example, learners can be encouraged to use AI feedback to identify recurring language problems, compare multiple revision options, request explanations rather than final answers, and evaluate whether a suggested expression fits the intended communicative purpose. The exploratory findings for anxiety and risk perception also suggest that students may experience affective tension or risk awareness while still reporting favorable acceptance evaluations. Anxiety should not be deliberately induced or treated as inherently desirable, but low-stakes practice, iterative revision, and reflective comparison may help students engage with AI feedback without excessive fear of evaluation. Similarly, risk awareness can be incorporated into students’ AI literacy by encouraging them to verify AI-generated content, protect personal information, avoid overreliance, and maintain academic integrity.

For curriculum designers, the findings suggest that AI-assisted English learning may be more useful when it is integrated into task sequences and learning progressions rather than treated as an optional or external add-on. Curriculum design can align AI-assisted activities with staged learning objectives, such as comprehension support, vocabulary development, writing revision, oral practice, feedback interpretation, and critical evaluation of AI-generated language. The descriptive group-related findings also suggest that a uniform curriculum model may not address all learners equally well. Low- and Intermediate-proficiency learners may benefit from more scaffolded feedback, guided practice, and explicit language support, whereas High-proficiency learners may require more advanced and autonomy-supportive tasks that go beyond surface-level correction. These proficiency-related implications should nevertheless be interpreted cautiously, particularly given the descriptive nature and smaller sample size of the High-proficiency subgroup (*n* = 30). Similarly, the observed differences between Humanities and STEM students should be interpreted as descriptive patterns requiring replication, not as evidence of fixed disciplinary traits. In addition, discipline-relevant English for Academic Purposes (EAP) or English for Specific Purposes (ESP) task examples may help students connect AI-assisted English learning with their academic and communicative needs, such as technical explanation, academic presentation, professional correspondence, text revision, or argument development.

For educational institutions, the findings suggest that effective AI-assisted English learning requires not only access to digital tools but also supportive pedagogical and governance conditions. Institutions may consider providing teacher professional development on AI-assisted language pedagogy, including task design, prompt literacy, feedback evaluation, academic integrity, and responsible AI use. Clear institutional guidelines may also help teachers and students understand appropriate boundaries for AI use in English learning, especially regarding privacy, data sharing, authorship, plagiarism, and assessment fairness. In addition, institutions should attend to equity of access and digital readiness, since students may differ in their confidence, prior exposure, and opportunities to receive guidance. A sustainable approach to AI-assisted English learning should therefore combine pedagogical scaffolding, critical AI literacy, ethical guidance, and context-sensitive curriculum design.

### 5.7 Concluding synthesis

In summary, this study suggests that university students’ acceptance of AI-assisted English learning tools is associated with a set of learner-related psychological, affective, and evaluative factors. Learning motivation and self-efficacy showed patterns consistent with the study’s theoretically informed expectations, while anxiety and risk perception showed exploratory positive associations that require cautious, context-sensitive interpretation. Mean-level differences by academic discipline and English proficiency suggest descriptive variation in average acceptance outcomes, and subgroup-specific regression models provide additional within-group association patterns. However, these subgroup findings should not be interpreted as formal evidence of between-group differences in regression coefficients.

These interpretations should also be read alongside the study’s statistical reporting choices and analytical boundaries. Robust standard errors were utilized to address heteroscedasticity in the full-sample adjusted models, whereas the subgroup-specific analyses were reported using exploratory ordinary OLS standard errors. In addition, as noted in the supplementary diagnostic checks, the regression findings should be interpreted strictly as descriptive, concurrent, and associative patterns rather than as causal, temporal, or mechanistic evidence. The study is nevertheless consistent with a cautious learner-centered account of AI-assisted English learning acceptance and points to the value of future studies that can examine temporal ordering, subgroup variation, and instructional conditions more directly. The specific limitations that qualify these conclusions are discussed next.

### 5.8 Limitations and future research directions

Several limitations should be considered when interpreting the findings of this study. These limitations do not invalidate the main findings, but they define the appropriate scope of inference and indicate directions for future research.

First, the study used a cross-sectional survey design, which limits causal and temporal interpretation. All reported associations reflect concurrent relationships observed at a single time point and should not be interpreted as causal, directional, or mechanistic effects. The regression analyses do not establish whether learning motivation, self-efficacy, anxiety, or risk perception preceded students’ acceptance evaluations, nor do they rule out possible reciprocal relationships. For example, favorable experiences with AI-assisted English learning tools may also be associated with stronger motivation, greater self-efficacy, or heightened awareness of affective and evaluative issues over time. Future longitudinal, panel-based, or carefully designed quasi-experimental research would be needed to examine how psychological correlates and acceptance outcomes develop across repeated engagement with AI-assisted English learning.

Second, the generalizability of the findings is constrained by the sampling context. The analytical sample was drawn from one public comprehensive university in Tianjin, northern China, and consisted of non-English-major second-year undergraduates enrolled in College English courses. Although this context is appropriate for examining AI-assisted English learning acceptance among students with relevant learning experience, the observed association patterns and group-related descriptive findings should not be assumed to generalize directly to other institutional types, regions, curricular arrangements, or learner populations. Future multi-institutional and cross-regional studies would help evaluate whether similar patterns are observed across different higher education contexts, AI implementation conditions, and English learning environments.

Third, the study relied on self-reported questionnaire data. Self-report measures are appropriate for assessing latent psychological and perceptual constructs such as perceived usefulness, perceived ease of use, satisfaction, behavioral intention, self-efficacy, motivation, anxiety, and risk perception. However, such measures may be influenced by subjective response tendencies, social desirability, recall limitations, and common-method bias. In addition, students’ reported perceptions and behavioral intentions may not fully correspond to actual AI-assisted English learning behaviors, such as frequency, depth of engagement, feedback interpretation, revision quality, or sustained use across tasks. Future studies could complement survey-based measures with behavioral indicators, learning analytics, usage logs, task-performance data, interviews, or classroom observations.

Fourth, the subgroup-specific regression analyses should be interpreted as exploratory and descriptive. These models were estimated separately within academic discipline and English proficiency subgroups to describe within-group association patterns, but they were not designed as formal tests of moderation or between-group coefficient differences. No group-by-correlate interaction tests, coefficient-comparison tests, or multi-group modeling procedures were conducted. Therefore, apparent differences in coefficient magnitude, statistical significance, or model fit across discipline or proficiency subgroups should not be interpreted as evidence that the associations statistically differed between learner groups. The subgroup-specific findings are better understood as preliminary patterns that may inform future hypothesis-driven research.

Fifth, the subgroup analyses were further limited by unequal subgroup sizes. This issue is particularly relevant for the High-proficiency subgroup, which included only 30 students. Estimates from this subgroup may be less stable and more sensitive to sampling variation than estimates from larger subgroups or from the full analytical sample. Accordingly, the High-proficiency results should be treated as tentative descriptions of the observed within-group patterns in this sample, rather than as reliable evidence about high-proficiency learners more broadly. Larger and more balanced proficiency subgroups are needed to examine whether these descriptive patterns are robust.

Sixth, risk perception showed comparatively weaker reliability and convergent validity evidence than the other constructs in the present sample. Although risk perception was included because concerns about privacy, reliability, dependency, and appropriate AI use are theoretically relevant to AI-assisted learning, its lower internal consistency and convergent validity evidence, specifically Cronbach’s alpha = 0.613, CR = 0.675, and AVE = 0.363, indicate that findings involving this construct should be interpreted with additional caution. This limitation does not require discarding the risk perception findings, but it suggests that they should be regarded as preliminary. Future research may refine the operationalization of risk perception by distinguishing among privacy risk, accuracy risk, dependency risk, academic-integrity concerns, and learner-autonomy concerns in AI-assisted English learning.

Finally, the study used different standard-error strategies for different analytical purposes. Robust standard errors were used in the full-sample adjusted regression models to support coefficient inference in light of heteroscedasticity diagnostics. By contrast, the subgroup-specific regression models used conventional OLS standard errors and were reported as exploratory descriptive evidence. This distinction should be kept in mind when interpreting the two sets of regression results. The full-sample and subgroup-specific models should not be treated as directly comparable tests of differential effects across groups.

## 6 Conclusion

This study examined university students’ acceptance of AI-assisted English language learning tools in a Chinese higher education context. Building on TAM-based acceptance outcomes, the study investigated how learner-related psychological correlates, specifically learning motivation, self-efficacy, anxiety, and risk perception, were associated with perceived usefulness, perceived ease of use, behavioral intention, and satisfaction. The findings showed that learning motivation and self-efficacy were recurring positive correlates of the four acceptance outcomes in the full analytical sample. Anxiety and risk perception also showed positive associations in the full-sample models, but these findings should be interpreted cautiously as exploratory concurrent patterns rather than as evidence that anxiety or perceived risk is beneficial. Mean-level comparisons and subgroup-specific regression analyses further suggested descriptive variation across academic discipline and English proficiency groups. These analyses did not provide formal evidence of between-group differences in regression coefficients.

Practical implications derived from these findings are discussed in section 5.6 Practical implications for AI-assisted English learning,” where suggestions are offered for language teachers, learners, curriculum designers, and educational institutions as pedagogical considerations rather than tested instructional recommendations. In brief, the findings suggest the potential value of guided AI use, critical feedback interpretation, proficiency-sensitive scaffolding, discipline-relevant task design, and institutional support for responsible and equitable AI-assisted English learning.

Several limitations qualify these conclusions. The study used a cross-sectional survey design, and all findings should therefore be interpreted as associative rather than causal, directional, or mechanistic. The analytical sample was drawn from a single public comprehensive university in Tianjin, northern China, which limits the generalizability of the findings to other institutional, regional, and curricular contexts. The study also relied on self-reported questionnaire data, which may be affected by subjective response tendencies and common-method bias. In addition, the subgroup-specific regression analyses were exploratory and descriptive, no interaction tests or formal coefficient-comparison tests were conducted, and the High-proficiency subgroup was relatively small. Finally, risk perception showed comparatively weaker reliability and convergent validity evidence in the present sample, so findings involving this construct should be interpreted with additional caution.

Future research can extend the present study in several directions. Longitudinal, panel-based, quasi-experimental, or experimental designs could examine the temporal ordering and possible reciprocal relationships between psychological correlates and acceptance outcomes. Multi-institutional and cross-regional studies with larger and more balanced subgroup samples would help assess whether the descriptive patterns observed here are stable across learner groups and higher education contexts. Future studies could also incorporate behavioral indicators, such as usage logs, learning analytics, task-performance data, or classroom observations, to complement self-reported perceptions. In addition, more refined measures of risk perception are needed to distinguish among privacy risk, accuracy risk, dependency risk, academic-integrity concerns, and learner-autonomy concerns in AI-assisted English learning. Overall, the study provides a restrained, learner-centered, and associational account of AI-assisted English learning acceptance, offering a basis for future research and context-sensitive pedagogical development rather than definitive causal or universally generalizable claims.

## Data Availability

The raw data supporting the conclusions of this article will be made available by the authors, without undue reservation.
