# Supplementary material for: Acceptance of AI-assisted English language learning tools in higher education: psychological correlates across disciplinary and proficiency groups
Source: Front Psychol. 2026 Jul 1;17:1806457. doi: 10.3389/fpsyg.2026.1806457 (PMC13368794; doi:10.3389/fpsyg.2026.1806457)
Supplement: Supplementary file 1 [file Data_Sheet_1.pdf]

Appendix A. Adapted Survey Items for AI-assisted English Learning

The following table lists the survey items used to measure the constructs in this study. Each item was measured on a 5-point Likert scale (1 = Strongly Disagree, 5 = Strongly Agree).

Construct	Source / reference	Adaptation focus	Item Code	Survey Item
Perceived Usefulness (PU)	Contextually adapted from Davis (1989)	Perceived usefulness of AI tools for English learning efficiency, resource access, and learning support	PU1	Using AI tools helps me improve the efficiency of completing my English learning tasks.
			PU2	Through AI tools, I can obtain the language learning resources I need more quickly.
			PU3	AI tools are a valuable resource for my English learning.
Perceived Ease of Use (PEOU)	Contextually adapted from Davis (1989)	Ease of learning, adapting to, and using AI tools for English learning	PEOU1	Learning how to use these AI tools is easy for me.
			PEOU2	I can quickly adapt to using AI tools in my English learning.
			PEOU3	Using AI tools for English learning is intuitive and not complicated.
Behavioral Intention (BI)	Contextually adapted from Venkatesh et al. (2012)	Continued and positive use intention toward AI-assisted English learning, including continued use and peer recommendation	BI1	I intend to continue using AI tools for learning English in the future.
			BI2	I would recommend using AI tools for English learning to my classmates and friends.
Self-Efficacy (SE)	Contextually adapted from the Computer Self-Efficacy scale (Compeau & Higgins, 1995)	Perceived capability to use AI tools to complete and manage English learning tasks	SE1	I know how to use AI tools to complete different types of English learning tasks.
			SE2	I am confident in flexibly applying various AI tools in my English learning.

Construct	Source / reference	Adaptation focus	Item Code	Survey Item
			SE3	I have a comprehensive understanding of how to use AI effectively for English learning.
			SE4	I am able to solve difficulties encountered when using AI tools to assist my learning on my own.
			SE5	I can use AI for English learning naturally and proficiently.
Learning Motivation (LM)	Contextually adapted with reference to the Academic Motivation Scale (Vallerand et al., 1992, 1993)	AI-related English learning motivation, including interest, active exploration, goal-oriented learning, task/exam reasons, feedback-driven improvement, and sustained effort	LM1	Using AI tools increases my interest in learning English.
			LM2	With the assistance of AI, I am more willing to actively explore new learning content.
			LM3	I use AI for learning because I believe it can help me achieve long-term language learning goals.
			LM4	I use AI because it helps me get good exam results or complete tasks assigned by teachers.
			LM5	Feedback from AI motivates me to continuously improve my English proficiency.
			LM6	Using AI strengthens my motivation to continue investing effort in English learning.
Anxiety (ANX)	Contextually adapted from the Artificial Intelligence Anxiety Scale (Wang & Wang, 2019)	Affective unease, worry, or reduced confidence associated with AI-assisted English learning	ANX1	I feel uneasy when I think my English progress may rely more on AI assistance than on my own effort.
			ANX2	I feel anxious when using AI for English

Construct	Source / reference	Adaptation focus	Item Code	Survey Item
				learning does not noticeably improve my language proficiency.
			ANX3	I feel anxious when I cannot judge whether the feedback provided by AI for my English learning is accurate or appropriate.
			ANX4	I feel nervous when I need to understand or adjust unfamiliar AI functions to complete English learning tasks.
			ANX5	Using AI for English learning makes me feel less confident about my English abilities.
			ANX6	I feel uneasy when I rely too much on AI to generate ideas for English learning tasks.
Risk Perception (RP)	Contextually adapted from Featherman & Pavlou (2003)	Cognitive appraisal of possible negative consequences of AI use, including inaccurate content, weakened language skills, reduced independent thinking, and privacy/data risks	RP1	I am concerned that AI-generated content may be inaccurate or misleading.
			RP2	I am concerned that using AI may weaken my English expression skills.
			RP3	I am concerned that over-reliance on AI tools may reduce my ability to think independently.
			RP4	I am concerned about potential risks to personal privacy or learning data posed by AI tools.
Satisfaction (SAT)	Contextually adapted from Bhattacherjee (2001)	Overall post-use evaluation and satisfaction with AI-assisted English learning	SAT1	Using AI tools makes my learning process more enjoyable and efficient.
			SAT2	I believe AI improves the efficiency and quality of my

Construct	Source / reference	Adaptation focus	Item Code	Survey Item
				learning.
			SAT3	Compared to traditional methods, AI-assisted learning better meets my personalized learning needs.
			SAT4	My experience with AI tools met my expectations for English learning.
			SAT5	Overall, I am satisfied with my experience of using AI for English learning.

Notes. Contextual adaptations mainly involved replacing the original technology-, system-, or computer-related wording with “AI tools” or “AI-assisted English learning.” In this appendix, “AI tools” refers to AI-based tools used by students to support English learning, whereas “AI-assisted English learning” refers to the learning context in which these tools were used. These wording changes were intended to retain the central construct meanings while aligning the items with the present research context. Anxiety items were interpreted as affective responses to AI-assisted English learning, whereas risk perception items were interpreted as cognitive appraisals of possible negative consequences. The learning motivation items were adapted to capture AI-related English learning motivation broadly, including interest, active exploration, goal-oriented learning, task- or exam-oriented reasons, feedback-driven improvement, and sustained effort. The present study did not examine separate motivation regulation subtypes.
