# Supplementary material for: Acceptance of AI-assisted English language learning tools in higher education: psychological correlates across disciplinary and proficiency groups
Source: Front Psychol. 2026 Jul 1;17:1806457. doi: 10.3389/fpsyg.2026.1806457 (PMC13368794; doi:10.3389/fpsyg.2026.1806457)
Supplement: Supplementary file 2 [file Data_Sheet_2.pdf]

Supplementary Tables S1–S7 provide additional statistical details supporting the main analyses, including full-sample regression outputs, diagnostic checks, correlation and validity evidence, post-hoc comparisons, and exploratory subgroup-specific regression models. The subgroup-specific regression models are reported for descriptive and exploratory purposes and should not be interpreted as formal tests of between-group differences in regression coefficients.

Supplementary Table S1a. Full-sample adjusted regression coefficients

Model ID	Outcome	Focal predictor	B	SE	β	t	p	95% CI for B	N
M1a	PU	Self-efficacy	0.363	0.037	0.614	9.69	< .001	[0.289, 0.437]	210
M1b	PU	Learning motivation	0.310	0.029	0.625	10.60	< .001	[0.252, 0.368]	210
M1c	PU	Risk perception	0.383	0.062	0.476	6.14	< .001	[0.260, 0.506]	210
M1d	PU	Anxiety	0.291	0.037	0.584	7.91	< .001	[0.219, 0.364]	210
M2a	PEOU	Self-efficacy	0.412	0.033	0.661	12.52	< .001	[0.348, 0.477]	210
M2b	PEOU	Learning motivation	0.350	0.028	0.669	12.58	< .001	[0.295, 0.405]	210
M2c	PEOU	Risk perception	0.401	0.065	0.472	6.15	< .001	[0.272, 0.529]	210
M2d	PEOU	Anxiety	0.281	0.039	0.535	7.16	< .001	[0.204, 0.359]	210
M3a	BI	Self-efficacy	0.254	0.029	0.605	8.81	< .001	[0.197, 0.311]	210
M3b	BI	Learning motivation	0.226	0.019	0.643	11.78	< .001	[0.188, 0.264]	210
M3c	BI	Risk perception	0.238	0.044	0.417	5.44	< .001	[0.152, 0.324]	210
M3d	BI	Anxiety	0.227	0.024	0.640	9.39	< .001	[0.179, 0.274]	210
M4a	Satisfaction	Self-efficacy	0.781	0.043	0.787	18.16	< .001	[0.700, 0.866]	210
M4b	Satisfaction	Learning motivation	0.674	0.035	0.810	19.29	< .001	[0.605, 0.743]	210
M4c	Satisfaction	Risk perception	0.566	0.105	0.419	5.39	< .001	[0.359, 0.773]	210
M4d	Satisfaction	Anxiety	0.451	0.062	0.539	7.23	< .001	[0.328, 0.574]	210

Note. Each row represents a separate adjusted linear regression model. All models were adjusted for gender, academic discipline, CET-4 score, and frequency of AI-assisted English learning tool use. B = unstandardized coefficient; SE = standard error; β = standardized coefficient; CI = confidence interval. SEs, t values, p values, and 95% CIs were based on robust standard errors. PU = perceived usefulness; PEOU = perceived ease of use; BI = behavioral intention.

Supplementary Table S1b. Full-sample adjusted regression model summaries

Model ID	Outcome	Focal predictor	R^2	F(df1, df2)	Model p	Cohen's f^2	N
M1a	PU	Self-efficacy	0.4427	F(6, 203) = 18.96	< .001	0.7944	210
M1b	PU	Learning motivation	0.4444	F(6, 203) = 24.08	< .001	0.7999	210
M1c	PU	Risk perception	0.2988	F(6, 203) = 11.63	< .001	0.4261	210
M1d	PU	Anxiety	0.3941	F(6, 203) = 14.65	< .001	0.6504	210
M2a	PEOU	Self-efficacy	0.5104	F(6, 203) = 35.83	< .001	1.0425	210
M2b	PEOU	Learning motivation	0.5069	F(6, 203) = 35.95	< .001	1.0280	210
M2c	PEOU	Risk perception	0.3066	F(6, 203) = 13.23	< .001	0.4422	210
M2d	PEOU	Anxiety	0.3562	F(6, 203) = 13.53	< .001	0.5533	210
M3a	BI	Self-efficacy	0.4552	F(6, 203) = 17.84	< .001	0.8355	210
M3b	BI	Learning motivation	0.4873	F(6, 203) = 32.46	< .001	0.9505	210
M3c	BI	Risk perception	0.2722	F(6, 203) = 13.28	< .001	0.3740	210
M3d	BI	Anxiety	0.4784	F(6, 203) = 24.23	< .001	0.9172	210
M4a	Satisfaction	Self-efficacy	0.6662	F(6, 203) = 70.65	< .001	1.9958	210
M4b	Satisfaction	Learning motivation	0.6807	F(6, 203) = 75.35	< .001	2.1319	210
M4c	Satisfaction	Risk perception	0.2471	F(6, 203) = 11.06	< .001	0.3282	210
M4d	Satisfaction	Anxiety	0.3442	F(6, 203) = 10.93	< .001	0.5249	210

Note. Each row corresponds to the same model listed in Supplementary Table S1a. Cohen's f^2 was calculated as $R^2 / (1 - R^2)$. PU = perceived usefulness; PEOU = perceived ease of use; BI = behavioral intention.

Supplementary Table S2. Multicollinearity diagnostics for the full-sample adjusted regression models

Model ID	Outcome	Focal predictor	VIF	Tolerance	Multicollinearity concern
M1a	PU	Self-efficacy	1.06	0.9448	No
M1b	PU	Learning motivation	1.08	0.9217	No
M1c	PU	Risk perception	1.06	0.9426	No
M1d	PU	Anxiety	1.10	0.9079	No
M2a	PEOU	Self-efficacy	1.06	0.9448	No
M2b	PEOU	Learning motivation	1.08	0.9217	No
M2c	PEOU	Risk perception	1.06	0.9426	No
M2d	PEOU	Anxiety	1.10	0.9079	No
M3a	BI	Self-efficacy	1.06	0.9448	No
M3b	BI	Learning motivation	1.08	0.9217	No
M3c	BI	Risk perception	1.06	0.9426	No
M3d	BI	Anxiety	1.10	0.9079	No
M4a	Satisfaction	Self-efficacy	1.06	0.9448	No
M4b	Satisfaction	Learning motivation	1.08	0.9217	No
M4c	Satisfaction	Risk perception	1.06	0.9426	No
M4d	Satisfaction	Anxiety	1.10	0.9079	No

Note. Model IDs correspond to the full-sample adjusted regression models reported in Supplementary Tables S1a and S1b. All models were adjusted for gender, academic discipline, CET-4 score, and frequency of AI-assisted English learning tool use. VIF = variance inflation factor. No serious multicollinearity concern was identified in the full-sample adjusted regression models.

Supplementary Table S3a. Overall diagnostic summary for the full-sample adjusted regression models

Diagnostic domain	Method or indicator	Result	Acceptable	Reporting implication
Residual normality	Q-Q plot and Shapiro-Wilk test	W = 0.9715, p < .001	No	Residuals did not fully satisfy the normality assumption; given N = 210, coefficient estimation was considered asymptotically acceptable, and results were interpreted cautiously.
Homoscedasticity	White test	Heteroscedasticity was detected in Models 1, 2, and 3, but not in Model 4	Partially	Robust standard errors were used to reduce the influence of heteroscedasticity on coefficient inference.
Use of robust standard errors	Robust standard errors	Robust standard errors were applied to all full-sample adjusted regression models.	Yes	SEs, t values, p values, and 95% CIs were based on robust standard errors.
Linearity / functional form	RESET test	Models 1–3 showed acceptable functional form; Model 4 showed slight specification bias	Partially	The main regression results were retained, but model diagnostics were reported transparently and results were interpreted as associative rather than causal.
Outliers / influential cases	Cook's distance, leverage values, and studentized residuals	No serious outliers or influential cases were identified	Yes	The regression results were considered broadly stable with respect to influential observations.
Overall diagnostic judgment	Combined review of residual, heteroscedasticity, functional-form, and influence diagnostics	The models showed some diagnostic limitations, especially non-normal residuals and heteroscedasticity, but robust SEs and influence diagnostics supported the stability of the reported associations	Generally acceptable with caution	The regression findings should be interpreted as association patterns rather than causal or mechanistic effects.

Note. Model 1 = perceived usefulness; Model 2 = perceived ease of use; Model 3 = behavioral intention; Model 4 = satisfaction. SE = standard error; CI = confidence interval.

Supplementary Table S3b. Heteroscedasticity and functional-form diagnostics

Model	Outcome	White test p	Homoscedasticity assumption	RESET p	Functional-form judgment	Robust SE used
Model 1	PU	0.0483	Marginal violation	0.7724	Acceptable	Yes
Model 2	PEOU	0.0001	Violated	0.7337	Acceptable	Yes
Model 3	BI	0.0000	Violated	0.5895	Acceptable	Yes
Model 4	Satisfaction	0.4682	Met	0.0059	Slight specification concern	Yes

Note. White test results indicated heteroscedasticity in Models 1–3, with Model 1 showing a marginal violation and Models 2–3 showing clearer violations. Robust standard errors were therefore used for coefficient inference across all full-sample adjusted regression models.

Supplementary Table S3c. Influence diagnostics for the full-sample adjusted regression models

Model ID	Outcome	Focal predictor	Maximum Cook's distance	Maximum leverage	Maximum absolute studentized residual	Flag in original output	N
M1a	PU	Self-efficacy	0.323	0.082	2.803	No	210
M1b	PU	Learning motivation	0.321	0.083	3.022	Yes	210
M1c	PU	Risk perception	0.435	0.088	2.985	No	210
M1d	PU	Anxiety	0.335	0.087	3.180	Yes	210
M2a	PEOU	Self-efficacy	0.196	0.082	3.021	Yes	210
M2b	PEOU	Learning motivation	0.196	0.083	3.241	Yes	210
M2c	PEOU	Risk perception	0.321	0.088	3.024	Yes	210
M2d	PEOU	Anxiety	0.267	0.087	3.159	Yes	210
M3a	BI	Self-efficacy	0.322	0.082	2.809	No	210
M3b	BI	Learning motivation	0.189	0.083	3.222	Yes	210
M3c	BI	Risk perception	0.366	0.088	2.750	No	210
M3d	BI	Anxiety	0.176	0.087	3.580	Yes	210
M4a	Satisfaction	Self-efficacy	0.152	0.082	3.375	Yes	210
M4b	Satisfaction	Learning motivation	0.141	0.083	3.585	Yes	210
M4c	Satisfaction	Risk perception	0.412	0.088	2.529	No	210
M4d	Satisfaction	Anxiety	0.297	0.087	2.843	No	210

Note. Model IDs correspond to the full-sample adjusted regression models reported in Supplementary Tables S1a and S1b. The flag in the original output reflects whether the diagnostic output identified a potential outlier or influential-case concern, mainly in relation to studentized residuals. Although several models showed maximum absolute studentized residuals slightly above 3, the maximum Cook's distance and leverage values did not indicate severe influential observations. These diagnostics therefore support retaining the full-sample regression results while interpreting the associations cautiously. PU = perceived usefulness; PEOU = perceived ease of use; BI = behavioral intention.

Supplementary Table S4. Pearson correlations among the main study variables and square roots of AVE

Variable	1	2	3	4	5	6	7	8
1. PU	0.872							
2. PEOU	0.869	0.893						
3. BI	0.796	0.769	0.855					
4. Satisfaction	0.723	0.710	0.725	0.858				
5. Self-efficacy	0.630	0.670	0.636	0.803	0.841			
6. Learning motivation	0.631	0.668	0.673	0.817	0.800	0.832		
7. Risk perception	0.496	0.500	0.447	0.447	0.488	0.479	0.603	
8. Anxiety	0.608	0.564	0.678	0.573	0.550	0.569	0.523	0.770

Note. Off-diagonal values are Pearson correlation coefficients. Diagonal values are the square roots of AVE. N = 210 for all correlations. All off-diagonal correlations were significant at $p < .001$. PU = perceived usefulness; PEOU = perceived ease of use; BI = behavioral intention; AVE = average variance extracted.

Supplementary Table S5. Tukey HSD post-hoc comparisons among English proficiency groups

Outcome	Pairwise comparison	Mean difference	SE	95% CI for mean difference	Adjusted p	Method
PU	Intermediate - Low	0.004	0.099	[-0.200, 0.193]	0.968	Tukey HSD
PU	Low - High	0.377	0.142	[0.097, 0.657]	0.009	Tukey HSD
PU	Intermediate - High	0.381	0.137	[0.110, 0.650]	0.006	Tukey HSD
PEOU	Intermediate - Low	-0.067	0.104	[-0.273, 0.140]	0.520	Tukey HSD
PEOU	Low - High	0.487	0.149	[0.193, 0.780]	0.001	Tukey HSD
PEOU	Intermediate - High	0.419	0.144	[0.137, 0.703]	0.004	Tukey HSD
BI	Intermediate - Low	0.024	0.108	[-0.185, 0.235]	0.822	Tukey HSD
BI	Low - High	0.220	0.154	[-0.085, 0.520]	0.154	Tukey HSD
BI	Intermediate - High	0.244	0.148	[-0.050, 0.535]	0.101	Tukey HSD
Satisfaction	Intermediate - Low	0.057	0.100	[-0.140, 0.256]	0.570	Tukey HSD
Satisfaction	Low - High	0.324	0.143	[0.042, 0.606]	0.025	Tukey HSD
Satisfaction	Intermediate - High	0.381	0.138	[0.108, 0.654]	0.006	Tukey HSD

Note. Mean differences, SEs, and 95% CIs are reported as item-average scores on a five-point scale to remain consistent with the main-text descriptive and group-difference tables. Values were transformed from the original sum-score Tukey HSD output by dividing by the number of items for each outcome: PU and PEOU = 3 items, BI = 2 items, and Satisfaction = 5 items. Adjusted p values and simultaneous 95% CIs were based on Tukey HSD post-hoc tests. Positive mean differences indicate that the first group in the pairwise comparison had a higher mean than the second group. Post-hoc comparisons for BI are reported for completeness only because the omnibus proficiency-group difference for BI was not statistically significant. PU = perceived usefulness; PEOU = perceived ease of use; BI = behavioral intention.

Supplementary Table S6a. Discipline-specific coefficient estimates

Subgroup	Model ID	Outcome	Focal predictor	B	SE	β	t	p	95% CI for B	N
STEM subgroup	M1a	PU	Self-efficacy	0.430	0.048	0.696	8.92	< .001	[0.334, 0.526]	91
STEM subgroup	M1b	PU	Learning motivation	0.373	0.041	0.710	9.09	< .001	[0.292, 0.455]	91
STEM subgroup	M1c	PU	Risk perception	0.468	0.084	0.621	5.57	< .001	[0.301, 0.635]	91
STEM subgroup	M1d	PU	Anxiety	0.316	0.054	0.649	5.87	< .001	[0.209, 0.423]	91
STEM subgroup	M2a	PEOU	Self-efficacy	0.479	0.050	0.723	9.53	< .001	[0.379, 0.579]	91
STEM subgroup	M2b	PEOU	Learning motivation	0.426	0.035	0.748	12.08	< .001	[0.356, 0.496]	91
STEM subgroup	M2c	PEOU	Risk perception	0.501	0.081	0.640	6.22	< .001	[0.341, 0.662]	91
STEM subgroup	M2d	PEOU	Anxiety	0.306	0.054	0.599	5.64	< .001	[0.198, 0.415]	91
STEM subgroup	M3a	BI	Self-efficacy	0.272	0.046	0.649	5.93	< .001	[0.181, 0.364]	91
STEM subgroup	M3b	BI	Learning motivation	0.255	0.030	0.715	8.53	< .001	[0.196, 0.315]	91
STEM subgroup	M3c	BI	Risk perception	0.307	0.059	0.587	5.18	< .001	[0.189, 0.425]	91
STEM subgroup	M3d	BI	Anxiety	0.220	0.038	0.657	5.73	< .001	[0.144, 0.296]	91
STEM subgroup	M4a	Satisfaction	Self-efficacy	0.785	0.066	0.843	11.88	< .001	[0.653, 0.916]	91
STEM subgroup	M4b	Satisfaction	Learning motivation	0.671	0.049	0.842	13.61	< .001	[0.573, 0.770]	91
STEM subgroup	M4c	Satisfaction	Risk perception	0.777	0.120	0.660	6.46	< .001	[0.538, 1.016]	91
STEM subgroup	M4d	Satisfaction	Anxiety	0.486	0.084	0.650	5.78	< .001	[0.319, 0.654]	91
Humanities subgroup	M1a	PU	Self-efficacy	0.338	0.046	0.567	7.37	< .001	[0.248, 0.429]	119
Humanities subgroup	M1b	PU	Learning motivation	0.264	0.037	0.567	7.11	< .001	[0.190, 0.337]	119
Humanities subgroup	M1c	PU	Risk perception	0.304	0.069	0.374	4.39	< .001	[0.166, 0.441]	119
Humanities subgroup	M1d	PU	Anxiety	0.266	0.040	0.535	6.65	< .001	[0.187, 0.345]	119
Humanities	M2a	PEOU	Self-efficacy	0.378	0.045	0.623	8.42	< .001	[0.289, 0.467]	119

Subgroup	Model ID	Outcome	Focal predictor	B	SE	β	t	p	95% CI for B	N
subgroup										
Humanities subgroup	M2b	PEOU	Learning motivation	0.295	0.036	0.624	8.09	< .001	[0.223, 0.367]	119
Humanities subgroup	M2c	PEOU	Risk perception	0.291	0.072	0.353	4.06	< .001	[0.149, 0.433]	119
Humanities subgroup	M2d	PEOU	Anxiety	0.257	0.042	0.509	6.16	< .001	[0.175, 0.340]	119
Humanities subgroup	M3a	BI	Self-efficacy	0.279	0.032	0.626	8.79	< .001	[0.216, 0.342]	119
Humanities subgroup	M3b	BI	Learning motivation	0.217	0.026	0.625	8.41	< .001	[0.166, 0.268]	119
Humanities subgroup	M3c	BI	Risk perception	0.195	0.052	0.322	3.75	< .001	[0.092, 0.298]	119
Humanities subgroup	M3d	BI	Anxiety	0.246	0.026	0.662	9.47	< .001	[0.194, 0.297]	119
Humanities subgroup	M4a	Satisfaction	Self-efficacy	0.807	0.062	0.760	13.09	< .001	[0.685, 0.929]	119
Humanities subgroup	M4b	Satisfaction	Learning motivation	0.657	0.048	0.795	13.74	< .001	[0.563, 0.752]	119
Humanities subgroup	M4c	Satisfaction	Risk perception	0.359	0.126	0.249	2.84	.005	[0.109, 0.609]	119
Humanities subgroup	M4d	Satisfaction	Anxiety	0.412	0.073	0.467	5.67	< .001	[0.268, 0.556]	119

Note. Each row represents a separate subgroup-specific adjusted linear regression model. Subgroup-specific regression models used ordinary OLS standard errors. SEs, t values, p values, and 95% CIs were based on conventional OLS estimation. These models are reported for exploratory and descriptive purposes only and should not be interpreted as formal tests of between-group differences in regression coefficients. B = unstandardized coefficient; SE = standard error; β = standardized coefficient; CI = confidence interval; PU = perceived usefulness; PEOU = perceived ease of use; BI = behavioral intention.

Supplementary Table S6b. Discipline-specific model summaries

Subgroup	Model ID	Outcome	Focal predictor	R^2	Adj. R^2	F (df1, df2)	Model p	Cohen's f^2	N
STEM subgroup	M1a	PU	Self-efficacy	0.5299	0.5023	F(5, 85) = 19.16	< .001	1.1272	91
STEM subgroup	M1b	PU	Learning motivation	0.5460	0.5139	F(5, 85) = 20.44	< .001	1.2026	91
STEM subgroup	M1c	PU	Risk perception	0.3946	0.3590	F(5, 85) = 11.08	< .001	0.6518	91
STEM subgroup	M1d	PU	Anxiety	0.4516	0.4194	F(5, 85) = 14.00	< .001	0.8235	91
STEM subgroup	M2a	PEOU	Self-efficacy	0.6024	0.5790	F(5, 85) = 25.76	< .001	1.5151	91
STEM subgroup	M2b	PEOU	Learning motivation	0.6427	0.6216	F(5, 85) = 30.57	< .001	1.7988	91
STEM subgroup	M2c	PEOU	Risk perception	0.4385	0.4054	F(5, 85) = 13.27	< .001	0.7809	91
STEM subgroup	M2d	PEOU	Anxiety	0.4312	0.3977	F(5, 85) = 12.89	< .001	0.7581	91
STEM subgroup	M3a	BI	Self-efficacy	0.4654	0.4340	F(5, 85) = 14.80	< .001	0.8706	91
STEM subgroup	M3b	BI	Learning motivation	0.5450	0.5182	F(5, 85) = 20.36	< .001	1.1978	91
STEM subgroup	M3c	BI	Risk perception	0.3669	0.3297	F(5, 85) = 9.85	< .001	0.5795	91
STEM subgroup	M3d	BI	Anxiety	0.4631	0.4315	F(5, 85) = 14.66	< .001	0.8625	91
STEM subgroup	M4a	Satisfaction	Self-efficacy	0.7200	0.7035	F(5, 85) = 43.71	< .001	2.5714	91
STEM subgroup	M4b	Satisfaction	Learning motivation	0.7244	0.7082	F(5, 85) = 44.68	< .001	2.6284	91
STEM subgroup	M4c	Satisfaction	Risk perception	0.4801	0.4496	F(5, 85) = 15.70	< .001	0.9234	91
STEM subgroup	M4d	Satisfaction	Anxiety	0.4876	0.4574	F(5, 85) = 16.18	< .001	0.9516	91
Humanities subgroup	M1a	PU	Self-efficacy	0.3457	0.3227	F(4, 114) = 15.06	< .001	0.5284	119
Humanities subgroup	M1b	PU	Learning motivation	0.3303	0.3068	F(4, 114) = 14.05	< .001	0.4932	119
Humanities	M1c	PU	Risk	0.1732	0.1442	F(4, 114) = 5.97	< .001	0.2095	119

Subgroup	Model ID	Outcome	Focal predictor	R^2	Adj. R^2	F (df1, df2)	Model p	Cohen's f^2	N
subgroup			perception						
Humanities subgroup	M1d	PU	Anxiety	0.3040	0.2796	F(4, 114) = 12.45	< .001	0.4368	119
Humanities subgroup	M2a	PEOU	Self-efficacy	0.3953	0.3741	F(4, 114) = 18.63	< .001	0.6537	119
Humanities subgroup	M2b	PEOU	Learning motivation	0.3770	0.3551	F(4, 114) = 17.25	< .001	0.6051	119
Humanities subgroup	M2c	PEOU	Risk perception	0.1428	0.1127	F(4, 114) = 4.75	.001	0.1666	119
Humanities subgroup	M2d	PEOU	Anxiety	0.2639	0.2381	F(4, 114) = 10.22	< .001	0.3585	119
Humanities subgroup	M3a	BI	Self-efficacy	0.4390	0.4193	F(4, 114) = 22.30	< .001	0.7825	119
Humanities subgroup	M3b	BI	Learning motivation	0.4192	0.3988	F(4, 114) = 20.57	< .001	0.7218	119
Humanities subgroup	M3c	BI	Risk perception	0.1625	0.1331	F(4, 114) = 5.53	< .001	0.1940	119
Humanities subgroup	M3d	BI	Anxiety	0.4732	0.4547	F(4, 114) = 25.60	< .001	0.8983	119
Humanities subgroup	M4a	Satisfaction	Self-efficacy	0.6269	0.6138	F(4, 114) = 47.88	< .001	1.6802	119
Humanities subgroup	M4b	Satisfaction	Learning motivation	0.6485	0.6361	F(4, 114) = 52.57	< .001	1.8450	119
Humanities subgroup	M4c	Satisfaction	Risk perception	0.1278	0.0972	F(4, 114) = 4.18	.003	0.1465	119
Humanities subgroup	M4d	Satisfaction	Anxiety	0.2718	0.2462	F(4, 114) = 10.64	< .001	0.3732	119

Note. Each row corresponds to the same model listed in Supplementary Table S6a. Model-level statistics were based on ordinary OLS regression models. Cohen's f^2 was calculated as $R^2 / (1 - R^2)$. These subgroup-specific models are reported for exploratory and descriptive purposes only and should not be interpreted as formal tests of between-group differences in regression coefficients.

Supplementary Table S7a. Proficiency-specific coefficient estimates

Subgroup	Model ID	Outcome	Focal predictor	B	SE	β	t	p	CI lower	CI upper	N
Low-proficiency subgroup	M1a	PU	Self-efficacy	0.400	0.058	0.646	6.91	< .001	0.284	0.514	77
Low-proficiency subgroup	M1b	PU	Learning motivation	0.302	0.056	0.574	5.39	< .001	0.190	0.413	77
Low-proficiency subgroup	M1c	PU	Risk perception	0.383	0.086	0.489	4.46	< .001	0.211	0.554	77
Low-proficiency subgroup	M1d	PU	Anxiety	0.224	0.052	0.463	4.28	< .001	0.120	0.329	77
Low-proficiency subgroup	M2a	PEOU	Self-efficacy	0.372	0.062	0.583	5.96	< .001	0.247	0.496	77
Low-proficiency subgroup	M2b	PEOU	Learning motivation	0.319	0.056	0.589	5.67	< .001	0.207	0.432	77
Low-proficiency subgroup	M2c	PEOU	Risk perception	0.391	0.088	0.483	4.45	< .001	0.215	0.566	77
Low-proficiency subgroup	M2d	PEOU	Anxiety	0.238	0.053	0.476	4.49	< .001	0.132	0.344	77
Low-proficiency subgroup	M3a	BI	Self-efficacy	0.280	0.040	0.632	7.09	< .001	0.201	0.359	77
Low-proficiency subgroup	M3b	BI	Learning motivation	0.224	0.037	0.594	5.98	< .001	0.149	0.300	77
Low-proficiency subgroup	M3c	BI	Risk perception	0.206	0.063	0.367	3.29	.002	0.081	0.331	77
Low-proficiency subgroup	M3d	BI	Anxiety	0.193	0.034	0.555	5.76	< .001	0.126	0.260	77
Low-proficiency subgroup	M4a	Satisfaction	Self-efficacy	0.792	0.066	0.801	12.06	< .001	0.661	0.923	77
Low-proficiency subgroup	M4b	Satisfaction	Learning motivation	0.664	0.065	0.790	10.29	< .001	0.536	0.793	77
Low-proficiency subgroup	M4c	Satisfaction	Risk perception	0.286	0.146	0.228	1.96	.054	-0.005	0.576	77
Low-proficiency subgroup	M4d	Satisfaction	Anxiety	0.376	0.078	0.485	4.79	< .001	0.220	0.533	77
Intermediate-proficiency subgroup	M1a	PU	Self-efficacy	0.357	0.045	0.629	7.86	< .001	0.267	0.448	103

Subgroup	Model ID	Outcome	Focal predictor	B	SE	β	t	p	CI lower	CI upper	N
Intermediate-proficiency subgroup	M1b	PU	Learning motivation	0.314	0.033	0.711	9.45	< .001	0.248	0.380	103
Intermediate-proficiency subgroup	M1c	PU	Risk perception	0.352	0.071	0.448	4.96	< .001	0.211	0.492	103
Intermediate-proficiency subgroup	M1d	PU	Anxiety	0.321	0.041	0.658	7.89	< .001	0.240	0.402	103
Intermediate-proficiency subgroup	M2a	PEOU	Self-efficacy	0.438	0.042	0.738	10.50	< .001	0.356	0.521	103
Intermediate-proficiency subgroup	M2b	PEOU	Learning motivation	0.356	0.032	0.771	11.08	< .001	0.292	0.419	103
Intermediate-proficiency subgroup	M2c	PEOU	Risk perception	0.338	0.076	0.412	4.45	< .001	0.187	0.488	103
Intermediate-proficiency subgroup	M2d	PEOU	Anxiety	0.250	0.049	0.489	5.14	< .001	0.153	0.346	103
Intermediate-proficiency subgroup	M3a	BI	Self-efficacy	0.271	0.034	0.628	7.86	< .001	0.203	0.340	103
Intermediate-proficiency subgroup	M3b	BI	Learning motivation	0.236	0.025	0.704	9.29	< .001	0.186	0.286	103
Intermediate-proficiency subgroup	M3c	BI	Risk perception	0.246	0.055	0.412	4.48	< .001	0.137	0.355	103
Intermediate-proficiency subgroup	M3d	BI	Anxiety	0.265	0.029	0.714	9.12	< .001	0.207	0.322	103
Intermediate-proficiency subgroup	M4a	Satisfaction	Self-efficacy	0.784	0.071	0.760	11.07	< .001	0.643	0.924	103
Intermediate-proficiency subgroup	M4b	Satisfaction	Learning motivation	0.699	0.046	0.873	15.36	< .001	0.609	0.789	103
Intermediate-proficiency subgroup	M4c	Satisfaction	Risk perception	0.608	0.132	0.427	4.62	< .001	0.347	0.869	103
Intermediate-proficiency subgroup	M4d	Satisfaction	Anxiety	0.438	0.085	0.494	5.18	< .001	0.270	0.606	103
High-proficiency subgroup	M1a	PU	Self-efficacy	0.374	0.104	0.671	3.60	.001	0.159	0.588	30
High-proficiency subgroup	M1b	PU	Learning motivation	0.376	0.083	0.696	4.55	< .001	0.206	0.547	30
High-proficiency subgroup	M1c	PU	Risk perception	0.526	0.180	0.546	2.92	.007	0.154	0.898	30
High-proficiency subgroup	M1d	PU	Anxiety	0.367	0.077	0.711	4.79	< .001	0.209	0.525	30
High-proficiency subgroup	M2a	PEOU	Self-efficacy	0.463	0.087	0.766	5.30	< .001	0.283	0.644	30
High-proficiency subgroup	M2b	PEOU	Learning motivation	0.408	0.076	0.695	5.37	< .001	0.251	0.565	30
High-proficiency subgroup	M2c	PEOU	Risk perception	0.573	0.174	0.548	3.29	.003	0.213	0.932	30
High-proficiency subgroup	M2d	PEOU	Anxiety	0.393	0.071	0.700	5.52	< .001	0.246	0.539	30
High-proficiency subgroup	M3a	BI	Self-efficacy	0.219	0.062	0.626	3.52	.002	0.091	0.347	30
High-proficiency subgroup	M3b	BI	Learning motivation	0.253	0.043	0.744	5.89	< .001	0.164	0.341	30
High-proficiency subgroup	M3c	BI	Risk perception	0.348	0.103	0.576	3.39	.002	0.136	0.560	30
High-proficiency subgroup	M3d	BI	Anxiety	0.230	0.043	0.708	5.31	< .001	0.140	0.319	30
High-proficiency subgroup	M4a	Satisfaction	Self-efficacy	0.784	0.083	0.917	9.39	< .001	0.612	0.956	30
High-proficiency subgroup	M4b	Satisfaction	Learning motivation	0.581	0.104	0.700	5.56	< .001	0.365	0.797	30
High-proficiency subgroup	M4c	Satisfaction	Risk perception	0.945	0.222	0.640	4.25	< .001	0.487	1.403	30
High-proficiency subgroup	M4d	Satisfaction	Anxiety	0.557	0.098	0.703	5.68	< .001	0.355	0.760	30

Note. Each row represents a separate subgroup-specific adjusted linear regression model. Subgroup-specific regression models used ordinary OLS standard errors. SEs, t values, p values, and 95% CIs were based on conventional OLS estimation. These models are reported for exploratory and descriptive purposes only and should not be interpreted as formal tests of between-group differences in regression coefficients. Results for the high-proficiency subgroup should be interpreted with particular caution because of the small subgroup size (n = 30). B = unstandardized coefficient; SE = standard error; β = standardized coefficient; CI = confidence interval; PU = perceived usefulness; PEOU = perceived ease of use; BI = behavioral intention.

Supplementary Table S7b. Proficiency-specific model summaries

Subgroup	Model ID	Outcome	Focal predictor	R²	Adj. R²	F	df1	df2	Model p	Cohen's f²	N
Low-proficiency subgroup	M1a	PU	Self-efficacy	0.4372	0.3889	9.06	6	70	< .001	0.7768	77
Low-proficiency subgroup	M1b	PU	Learning motivation	0.3316	0.2744	5.79	6	70	< .001	0.4961	77
Low-proficiency subgroup	M1c	PU	Risk perception	0.2629	0.1998	4.16	6	70	.001	0.3567	77
Low-proficiency subgroup	M1d	PU	Anxiety	0.2503	0.1860	3.89	6	70	.002	0.3339	77
Low-proficiency subgroup	M2a	PEOU	Self-efficacy	0.3843	0.3315	7.28	6	70	< .001	0.6242	77
Low-proficiency subgroup	M2b	PEOU	Learning motivation	0.3643	0.3098	6.69	6	70	< .001	0.5731	77
Low-proficiency subgroup	M2c	PEOU	Risk perception	0.2766	0.2146	4.46	6	70	< .001	0.3824	77
Low-proficiency subgroup	M2d	PEOU	Anxiety	0.2798	0.2181	4.53	6	70	< .001	0.3885	77
Low-proficiency subgroup	M3a	BI	Self-efficacy	0.4882	0.4444	11.13	6	70	< .001	0.9539	77
Low-proficiency subgroup	M3b	BI	Learning motivation	0.4184	0.3685	8.39	6	70	< .001	0.7194	77
Low-proficiency subgroup	M3c	BI	Risk perception	0.2384	0.1732	3.65	6	70	.003	0.3130	77
Low-proficiency subgroup	M3d	BI	Anxiety	0.4036	0.3525	7.89	6	70	< .001	0.6767	77
Low-proficiency subgroup	M4a	Satisfaction	Self-efficacy	0.7164	0.6921	29.48	6	70	< .001	2.5261	77
Low-proficiency subgroup	M4b	Satisfaction	Learning motivation	0.6527	0.6229	21.92	6	70	< .001	1.8794	77
Low-proficiency subgroup	M4c	Satisfaction	Risk perception	0.1726	0.1017	2.43	6	70	.034	0.2086	77
Low-proficiency subgroup	M4d	Satisfaction	Anxiety	0.3429	0.2866	6.09	6	70	< .001	0.5218	77
Intermediate-proficiency subgroup	M1a	PU	Self-efficacy	0.4229	0.3931	14.22	5	97	< .001	0.7328	103
Intermediate-proficiency subgroup	M1b	PU	Learning motivation	0.5085	0.4831	20.07	5	97	< .001	1.0346	103
Intermediate-proficiency subgroup	M1c	PU	Risk perception	0.2465	0.2077	6.35	5	97	< .001	0.3271	103
Intermediate-proficiency subgroup	M1d	PU	Anxiety	0.4247	0.3950	14.32	5	97	< .001	0.7382	103
Intermediate-proficiency subgroup	M2a	PEOU	Self-efficacy	0.5550	0.5321	24.20	5	97	< .001	1.2472	103
Intermediate-proficiency subgroup	M2b	PEOU	Learning motivation	0.5806	0.5589	26.85	5	97	< .001	1.3844	103
Intermediate-proficiency subgroup	M2c	PEOU	Risk perception	0.2105	0.1698	5.17	5	97	< .001	0.2666	103
Intermediate-proficiency subgroup	M2d	PEOU	Anxiety	0.2532	0.2147	6.58	5	97	< .001	0.3390	103
Intermediate-proficiency subgroup	M3a	BI	Self-efficacy	0.4252	0.3956	14.35	5	97	< .001	0.7397	103
Intermediate-proficiency subgroup	M3b	BI	Learning motivation	0.5017	0.4760	19.53	5	97	< .001	1.0068	103
Intermediate-proficiency subgroup	M3c	BI	Risk perception	0.2203	0.1801	5.48	5	97	< .001	0.2825	103
Intermediate-proficiency subgroup	M3d	BI	Anxiety	0.4935	0.4674	18.90	5	97	< .001	0.9743	103
Intermediate-proficiency subgroup	M4a	Satisfaction	Self-efficacy	0.5754	0.5535	26.29	5	97	< .001	1.3552	103
Intermediate-proficiency subgroup	M4b	Satisfaction	Learning motivation	0.7202	0.7058	49.94	5	97	< .001	2.5740	103
Intermediate-proficiency subgroup	M4c	Satisfaction	Risk perception	0.2124	0.1718	5.23	5	97	< .001	0.2697	103
Intermediate-proficiency subgroup	M4d	Satisfaction	Anxiety	0.2475	0.2087	6.38	5	97	< .001	0.3289	103
High-proficiency subgroup	M1a	PU	Self-efficacy	0.5196	0.4195	5.19	5	24	.002	1.0816	30
High-proficiency subgroup	M1b	PU	Learning motivation	0.6030	0.5203	7.29	5	24	< .001	1.5189	30

Subgroup	Model ID	Outcome	Focal predictor	R^2	Adj. R^2	F	df1	df2	Model p	Cohen's f^2	N
High-proficiency subgroup	M1c	PU	Risk perception	0.4536	0.3397	3.98	5	24	.009	0.8302	30
High-proficiency subgroup	M1d	PU	Anxiety	0.6220	0.5432	7.90	5	24	< .001	1.6455	30
High-proficiency subgroup	M2a	PEOU	Self-efficacy	0.7108	0.6505	11.80	5	24	< .001	2.4578	30
High-proficiency subgroup	M2b	PEOU	Learning motivation	0.7146	0.6552	12.02	5	24	< .001	2.5039	30
High-proficiency subgroup	M2c	PEOU	Risk perception	0.5670	0.4768	6.29	5	24	< .001	1.3095	30
High-proficiency subgroup	M2d	PEOU	Anxiety	0.7235	0.6659	12.56	5	24	< .001	2.6166	30
High-proficiency subgroup	M3a	BI	Self-efficacy	0.5626	0.4715	6.17	5	24	< .001	1.2862	30
High-proficiency subgroup	M3b	BI	Learning motivation	0.7290	0.6725	12.91	5	24	< .001	2.6900	30
High-proficiency subgroup	M3c	BI	Risk perception	0.5517	0.4583	5.91	5	24	.001	1.2306	30
High-proficiency subgroup	M3d	BI	Anxiety	0.6951	0.6316	10.95	5	24	< .001	2.2798	30
High-proficiency subgroup	M4a	Satisfaction	Self-efficacy	0.8679	0.8404	31.54	5	24	< .001	6.5700	30
High-proficiency subgroup	M4b	Satisfaction	Learning motivation	0.7303	0.6741	13.00	5	24	< .001	2.7078	30
High-proficiency subgroup	M4c	Satisfaction	Risk perception	0.6481	0.5748	8.84	5	24	< .001	1.8417	30
High-proficiency subgroup	M4d	Satisfaction	Anxiety	0.7366	0.6817	13.42	5	24	< .001	2.7965	30

Note. Each row corresponds to the same model listed in Supplementary Table S7a. Model-level statistics were based on ordinary OLS regression models. Cohen's f^2 was calculated as $R^2 / (1 - R^2)$. Results for the High-proficiency subgroup should be interpreted with particular caution because of the small subgroup size (n = 30). PU = perceived usefulness; PEOU = perceived ease of use; BI = behavioral intention.
